# Trichloroethylene and Cancer: Systematic and Quantitative Review of Epidemiologic Evidence for Identifying Hazards

**DOI:** 10.3390/ijerph8114238

**Published:** 2011-11-09

**Authors:** Cheryl Siegel Scott, Jennifer Jinot

**Affiliations:** National Center for Environmental Assessment, Office of Research and Development, United States Environmental Protection Agency, 1200 Pennsylvania Avenue, Washington, DC 20460, USA; E-Mail: Jinot.Jennifer@epa.gov

**Keywords:** trichloroethylene, meta-analysis, kidney cancer, liver cancer, NHL, occupational exposure

## Abstract

We conducted a meta-analysis focusing on studies with high potential for trichloroethylene (TCE) exposure to provide quantitative evaluations of the evidence for associations between TCE exposure and kidney, liver, and non-Hodgkin lymphoma (NHL) cancers. A systematic review documenting essential design features, exposure assessment approaches, statistical analyses, and potential sources of confounding and bias identified twenty-four cohort and case-control studies on TCE and the three cancers of interest with high potential for exposure, including five recently published case-control studies of kidney cancer or NHL. Fixed- and random-effects models were fitted to the data on overall exposure and on the highest exposure group. Sensitivity analyses examined the influence of individual studies and of alternative risk estimate selections. For overall TCE exposure and kidney cancer, the summary relative risk (RRm) estimate from the random effects model was 1.27 (95% CI: 1.13, 1.43), with a higher RRm for the highest exposure groups (1.58, 95% CI: 1.28, 1.96). The RRm estimates were not overly sensitive to alternative risk estimate selections or to removal of an individual study. There was no apparent heterogeneity or publication bias. For NHL, RRm estimates for overall exposure and for the highest exposure group, respectively, were 1.23 (95% CI: 1.07, 1.42) and 1.43 (95% CI: 1.13, 1.82) and, for liver cancer, 1.29 (95% CI: 1.07, 1.56) and 1.28 (95% CI: 0.93, 1.77). Our findings provide strong support for a causal association between TCE exposure and kidney cancer. The support is strong but less robust for NHL, where issues of study heterogeneity, potential publication bias, and weaker exposure-response results contribute uncertainty, and more limited for liver cancer, where only cohort studies with small numbers of cases were available.

## 1. Introduction

The interpretation of the epidemiologic studies on cancer and trichloroethylene (TCE) continues to be an area of considerable interest despite numerous reviews, including those of multidisciplinary expert panels whose conclusions have ranged widely due, in part, to differences in the qualitative evaluation of the epidemiologic data as well as in the studies available at the time [1–5]. Two advisory panels reviewing the epidemiologic evidence on cancer and TCE recommended meta-analysis as an approach to synthesize the data, noting individual studies had limited statistical power for relatively uncommon cancers such as kidney, liver, and non-Hodgkin lymphoma (NHL) [6,7].

In this paper, we present findings from a systematic review and a comprehensive meta-analysis of studies of occupational TCE exposure and cancer, focusing on studies with high potential for TCE exposure and following guidance for epidemiologic study reporting and meta-analysis practice [8–10]. We focus on three specific cancers of *a priori* interest from rodent bioassays of TCE exposure [11–13] and a broader qualitative review of the epidemiologic data—kidney cancer, liver cancer, and NHL. We consider current disease classifications for NHL and carry out a systematic evaluation of the literature. Our meta-analysis updates the literature covered by previous meta-analyses of TCE exposure and cancer [14–19], adding four case-control studies on NHL [20–23], one case-control study on renal cell carcinoma [24], two studies in a cohort of aerospace workers [25,26], and an updated mortality follow-up of a cohort of aircraft maintenance workers [27]. The incorporation of clear *a priori* guidelines for identifying studies with moderate-to-high probability of TCE exposure, inclusion of both cohort and case-control studies, supplemental examination of the highest exposure group in each study to reduce the impact of exposure misclassification, and assessments of heterogeneity and sensitivity provide insight for the evaluation of a causal link between TCE and these specific cancers.

## 2. Methods

A thorough search of the literature was carried out without restriction on year of publication or language to identify all studies that assessed the relationship between cancer and TCE following these approaches: a search of the bibliographic databases PubMed (http://www.ncbi.nlm.nih.gov/pubmed/), TOXNET (http://toxnet.nlm.nih.gov/) and EMBASE (http://www.embase.com/) using the terms “trichloroethylene cancer epidemiology” and ancillary terms, “degreasers,” “aircraft, aerospace or aircraft maintenance workers,” “metal workers,” and “electronic workers,” “trichloroethylene and cohort,” or, “trichloroethylene and case-control;” examination of bibliographies of reviews of the TCE epidemiologic literature such as those of the Institute of Medicine [28], National Research Council [5,6] and Scott and Chiu [4]; and review of bibliographies of individual studies and previous meta-analyses for relevant studies. Only studies in press or published in scientific journals, as of December 2010, or their additional analyses provided through personnel communication with the authors were considered. Studies with multiple published analyses based on updates to the same cohort are identified by the most recent publication.

### 2.1. Study Selection and Data Extraction

Studies selected for inclusion in the meta-analysis met the following criteria: (1) cohort or case-control design; (2) exposed and control groups in cohort studies and cases and controls in case-control studies are comparable and drawn from the same base population; (3) TCE exposure potential and some estimate of TCE exposure assessed for each subject by reference to industrial hygiene records, individual biomarkers, job-exposure matrices, expert assessment, water distribution models, or questionnaire responses (case-control studies); and (4) relative risk (RR) estimates for kidney cancer, liver cancer, or NHL.

The general approach for selecting RR estimates and associated confidence intervals (CIs) was to pick a single RR estimate for overall TCE exposure *versus* no TCE exposure. When multiple estimates were available for the same study based on different subcohorts with different inclusion criteria, the preference for overall exposure was to select the RR estimate that represented the largest population in the study, while trying to minimize the likelihood of TCE exposure misclassification. A subcohort with more restrictive inclusion criteria was selected if the goal for the definition of the subcohort was to reduce exposure misclassification (e.g., including only subjects with more probable TCE exposure) but not if the goal was to reflect subjects with greater exposure (e.g., routine *versus* any exposure). When available, RR estimates from internal analyses were chosen over standardized incidence or mortality ratios (SIRs, SMRs), and adjusted RR estimates were selected over crude estimates. Odds ratios in case-control studies were considered to approximate the RR, or more specifically the rate ratio, as the cancers of interest are rare diseases in both the exposed and unexposed groups, with lifetime risks considerably less than 10% [29]. No correction was made to cause-specific mortality estimates based on an overall standardized mortality ratio to adjust for the healthy worker bias. In separate analyses, a RR estimate for the highest exposure group was selected, with a preference for cumulative exposure if available; however, often duration was the sole exposure metric presented.

### 2.2. Statistical Methods

Random-effects models were used for the primary analyses, employing the methodology of DerSimonian and Laird [30], with fixed-effect analyses conducted for comparison. Both approaches combine effect measures (in this case, RR estimates) weighted by the inverse variance; however, the random-effects model takes estimates of between-study, as well as within-study, variation into account. The meta-analysis calculations are based on natural-logarithm-transformed relative risk (log RR) values. For the weights, either an estimate of the standard error (SE) of the log RR, from which to estimate the variance, was obtained from the CIs of the RR estimate [31] or, for SMRs and SIRs, an estimate of the variance of the log RR was calculated directly as the inverse of the observed number of deaths (or cases) [32]. In some case-control studies, no overall odds ratio (OR) was reported and a crude OR estimate was used, with the variance of the log OR estimated in accordance with the method proposed by Woolf [33], as described by Breslow and Day [34]. All analyses were performed using Microsoft Excel^®^ spreadsheets and the software package Comprehensive Meta-Analysis, Version 2 (© 2006, Biostat, Inc.); forest plots were created using SAS, Version 9.2 (© 2002–2008, SAS Institute, Inc.). We calculated summary meta-relative-risk (RRm) estimates for overall TCE exposure and for the highest exposure groups in studies that provided data by exposure group. Examination of the highest exposure group was undertaken not to provide an estimate of risk associated with a specific exposure level, as exposure assessments differed greatly in quality and in the exposure metrics used, but, rather, to identify subjects with the greatest potential for TCE exposure, reducing potential bias from exposure misclassification. Additionally, we conducted analyses of the sensitivity of the summary estimates to alternative study inclusion selections or to alternative selections of a study’s RR estimate and influence analyses to examine the impact of individual studies on the summary estimates. Heterogeneity was assessed using the *Q*-statistic [30] and the *I^2^*-value [35]. Publication bias was assessed a number of ways, including funnel plots, the “trim and fill” procedure of Duval and Tweedie [36], forest plots of studies sorted by the SE, and cumulative meta-analyses after sorting studies by the SE.

## 3. Results

Twenty-four studies met the inclusion criteria and were eligible for meta-analysis. Table 1 lists the literature reference, study design, number of subjects, follow-up period, exposure assessment approach and whether RRs were based on incidence or on mortality. Eleven cohort studies were identified, four European studies of cancer incidence in degreasers from predominantly iron and metal industries [37–40] and seven studies in the United States of mortality in electrical, aerospace or aircraft maintenance workers [26,27,41–43] or of both incidence and mortality in aircraft maintenance and aerospace workers [25,44]. The study periods and base populations in Zhao *et al*. [25] and Boice *et al.* [26] overlap, although the studies differ in their exposure assessment approach. Zhao *et al.* [25] is preferred for the primary analysis given its larger number of TCE-exposed subjects, larger number of kidney cancer and NHL deaths, semi-quantitative exposure assessment, and internal referent group; Boice *et al*. [26] was used in the sensitivity analyses. Radican *et al*. [27] updated mortality in the National Cancer Institute (NCI) cohort of Blair *et al*. [44] and is preferred in the main analysis, given its additional 10 years of follow-up; the incidence findings reported in the earlier publication by Blair *et al.* [44] were used in the sensitivity analyses. The analysis treats Greenland *et al.* [42], a case-control study of multiple cancer sites nested within an occupational cohort, as a cohort study because the OR was estimated from incidence density sampling [45]. Of the thirteen case-control studies, seven were of NHL [20–23,46–48], five of renal cell carcinoma [24,49–52] and one of multiple cancer sites that included NHL and renal cell carcinoma [53]. No case-control studies of primary liver cancer or liver and biliary cancers were identified which fulfilled the inclusion criteria.

A listing of studies not meeting the selection criteria and the reasons for the exclusions can be found in the Supplemental Material.

### 3.1. Kidney Cancer

**Figure 1:**
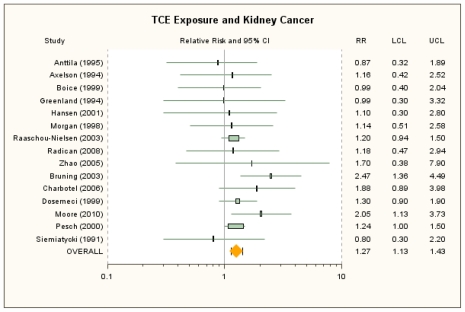
Forest plot of overall TCE exposure and kidney cancer from random-effects meta-analysis. Individual study results are plotted with 95% confidence intervals. Symbol sizes reflect relative weights of the 15 cohort and case-control studies.

Primary and alternate RR estimates for kidney cancer from the individual cohort and case-control studies are presented in Table 2, and a forest plot of the primary RR estimates and the summary RRm estimate for overall exposure is displayed in Figure 1. The RRm from the primary random-effects meta-analysis of the 15 studies was 1.27 (95% CI: 1.13, 1.43) and was identical to the RRm from the fixed-effect model. The studies of Pesch *et al*. [52], Raaschou-Nielsen *et al*. [40], and Dosemeci *et al*. [51] contributed about 75% of the weight, although no single study was overly influential and removal of individual studies resulted in RRm estimates that were all statistically significant and that ranged from 1.24 (removing Brüning *et al*. [49]) to 1.30 (removing Raaschou-Nielsen *et al*. [40]). Similarly, the RRm estimate was not highly sensitive to alternate RR estimate selections. Use of the 13 alternate RR selections, individually, resulted in RRm estimates that ranged from 1.21 (95% CI: 1.09, 1.34) to 1.32 (95% CI: 1.17, 1.49) (see Supplemental Material).

There was no observable heterogeneity across the studies of overall exposure (*I^2^* = 0%). Nevertheless, subgroup analyses examining cohort and case-control studies separately were carried out as sensitivity analyses. The random-effects model yielded RRm estimates of 1.16 (95% CI: 0.96, 1.40) for the cohort studies and 1.48 (1.15, 1.91) for the case-control studies, with RRm estimates not statistically significant different (p = 0.12) between cohort or case-control studies. No heterogeneity was observed in the cohort subgroup (*I^2^* = 0%), and low-to-moderate heterogeneity in the case-control subgroup was suggested by the *I^2^*-value of 41% (p = 0.14 for heterogeneity). No evidence of publication bias was observed in the data set for kidney cancer and overall TCE exposure. The trim-and-fill procedure determined that there was no imbalance in the funnel plot indicative of publication bias (see Supplemental Material).

**Figure 2:**
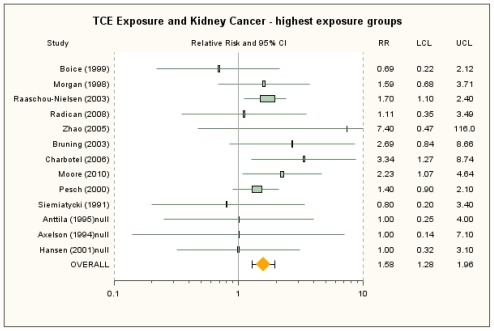
Forest plot of highest TCE exposure group and kidney cancer from random-effects meta-analysis. Individual study results are plotted with 95% confidence intervals. A risk estimate of 1.0 is assigned for highest exposure in Anttila *et al*. [38], Axelson *et al*. [37] and Hansen *et al*. [39] to account for presumed reporting bias. Symbol sizes reflect relative weights of the 13 cohort and case-control studies.

For the highest exposure groups, the primary random-effects meta-analysis, in which null estimates (RR = 1.0) were included for three studies which reported exposure-group results for select other cancers but not for kidney cancer, to address ostensible reporting bias, yielded an RRm estimate of 1.58 (95% CI: 1.28, 1.96) (see Figure 2) [37–39]. In an analysis of only the ten studies reporting results by exposure level, the RRm estimate was 1.64 (95% CI: 1.31, 2.04), similar to the RRm from the primary analysis. No single study was overly influential, as removal of individual studies resulted in RRm estimates that were all statistically significant and that ranged from 1.52 (removing Raaschou-Nielsen *et al*. [40]) to 1.64 (removing Pesch *et al*. [52]). Similarly, the RRm estimate was not highly sensitive to alternate RR estimate selections. Use of the 18 alternate selections, individually, resulted in RRm estimates that ranged from 1.47 (95% CI: 1.20, 1.79) to 1.60 (95% CI: 1.29, 1.98), with most alternate selections yielding RRm estimates in the narrow range 1.54–1.60 (see Supplemental Material). There was no observable heterogeneity across the studies for any of the analyses conducted with the highest exposure groups other than a negligible amount of heterogeneity observed in the sensitivity analysis with the Pesch *et al*. [52] alternate RR estimate (*I*^2^ = 0.64%).

### 3.2. Liver Cancer

**Figure 3:**
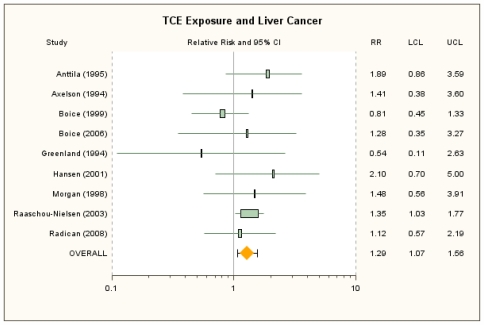
Forest plot of overall TCE exposure and liver cancer from random-effects meta-analysis. Individual study results are plotted with 95% confidence intervals. Symbol sizes reflect relative weights of the 9 cohort studies.

The RRm estimate from the random-effects meta-analysis of the nine independent cohort studies of overall TCE exposure and liver and gall bladder/biliary passages was 1.29 (95% CI: 1.07, 1.56) (Figure 3), identical to that from the fixed-effect model. Individual study RR estimates and alternatives are in Table 3. Relative risk estimates in these studies were generally based on fewer than 10 events. The number of events in Raaschou-Nielsen *et al*. [40] was over 2-fold higher than the numbers in other studies and contributed about 53% of the total weight. The RRm estimate decreases somewhat without this large study and, as expected, has less precision (RRm = 1.22; 95% CI: 0.93, 1.61). The influence analysis showed no other single study was overly influential; removal of any of the other individual studies resulted in RRm estimates that were all statistically significant and that ranged from 1.24 (removing Anttila *et al*. [38]) to 1.39 (removing Boice *et al*. [41]). The RRm, furthermore, was not highly sensitive to the seven alternate RR estimate selections, nor to the simultaneous substitution of results for liver cancer alone for the three studies for which these were available, which yielded a RRm of 1.25 (95% CI: 0.99, 1.57) (see Supplemental Material).

Analysis of the nine studies of overall exposure revealed no apparent heterogeneity (*I^2^*-values were 0%). Since all studies on liver cancer were of cohort design, no analyses were conducted examining cohort and case-control studies separately. Funnel plots and other tests performed to examine potential publication bias in the TCE liver cancer data set did not find any evidence of missing studies or of a relationship between RR estimate and study size (see Supplemental Material).

**Figure 4:**
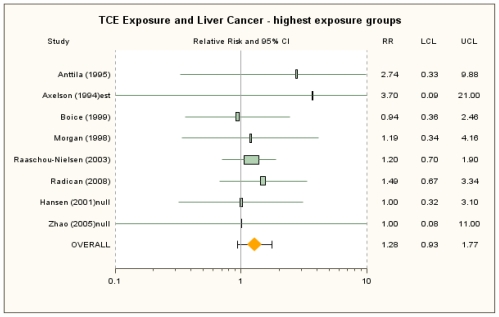
Forest plot of highest TCE exposure group and liver cancer from random-effects meta-analysis. Individual study results are plotted with 95% confidence intervals. A risk estimate of 1.0 is assigned for highest exposure in Hansen *et al*. [39] and Zhao *et al*. [25] to account for presumed reporting bias. Symbol sizes reflect relative weights of the 8 cohort studies.

The RRm estimate from the random-effects meta-analysis of the six studies presenting results for highest exposure groups was 1.32 (95% CI: 0.93, 1.86). The RRm estimate from the primary random-effects meta-analysis with null RR estimates (*i.e.*, 1.0) included for Hansen *et al*. [39] and Zhao *et al*. [25] to address presumed reporting bias was 1.28 (95% CI: 0.93, 1.77) (Figure 4). The RRm estimate for liver cancer in the highest exposure groups was lower than that for overall exposure and primarily reflects the lower observed RR estimate for the highest exposure group in Raaschou-Nielsen *et al*. [40], the study carrying the greatest weight. No other single study was overly influential, and the RRm estimate was not sensitive to alternate RR selections (see Supplemental Material). There was no observable heterogeneity across the eight studies for any of the analyses conducted with the highest exposure groups (*I^2^*-values were 0%).

### 3.3. Non-Hodgkin Lymphoma

**Figure 5:**
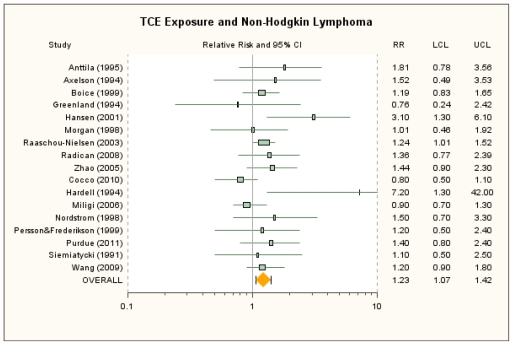
Forest plot of overall TCE exposure and non-Hodgkin lymphoma from random-effects meta-analysis. Individual study results are plotted with 95% confidence intervals. Symbol sizes reflect relative weights of the 17 cohort and case-control studies.

Primary and alternate RR estimates for NHL in the nine cohort and eight case-control studies are presented in Table 4, and a forest plot for overall exposure is displayed in Figure 5. The RRm estimate from the primary random-effects meta-analysis of overall exposure was 1.23 (95% CI: 1.07, 1.42). This RRm estimate was not overly sensitive to removal of individual studies, with resulting RRm estimates that were all statistically significant and that ranged from 1.18 (removing Hansen *et al*. [39]) to 1.27 (removing Miligi *et al*. [20] or Cocco *et al*. [21]). Removal of Hardell *et al*. [46], whose RR estimate of 7.2 is a relative outlier, only decreased the RRm estimate to 1.21 (95% CI: 1.07, 1.38), since this study contributes little weight to the meta-analysis. Similarly, the primary RRm estimate was not highly sensitive to the alternate RR estimate selections, with which RRm estimates ranged from 1.20 (95% CI: 1.03, 1.39) to 1.28 (95% CI: 1.09, 1.49); nor was it sensitive to restriction of the analysis to the 13 studies for which RR estimates for the traditional definition of NHL were available (RRm = 1.27, 95% CI: 1.05, 1.55) (see Supplemental Material). Low-to-moderate heterogeneity was observed in the primary analysis of overall exposure (*I^2^*-value was 26%; *p* = 0.16) and in each of the meta-analyses with alternative RR selections (*I^2^* = 25% to 34%; *p* = 0.09 to 0.17). Subgroup analyses examined cohort and case-control studies and overall exposure separately to investigate the heterogeneity. In cohort studies, the RRm was 1.33 (95% CI: 1.13, 1.58) and, in case-control studies, 1.11 (95% CI: 0.89, 1.38). The subgroup RRm estimates were not statistically significantly different (*p* = 0.19, under the random-effects model). There was evidence of potential publication bias, as the funnel plot appeared asymmetrical and suggested some relationship between RR estimate and study size (see Supplemental Material). Using Duval and Tweedie’s trim-and-fill procedure to counter-balance the apparent asymmetry of the more extreme values in the funnel plot yielded a RRm estimate of 1.15 (95% CI: 0.97, 1.36). Thus, if there is publication bias in this data set, it does not appear to account completely for the finding of an increased NHL risk.

**Figure 6:**
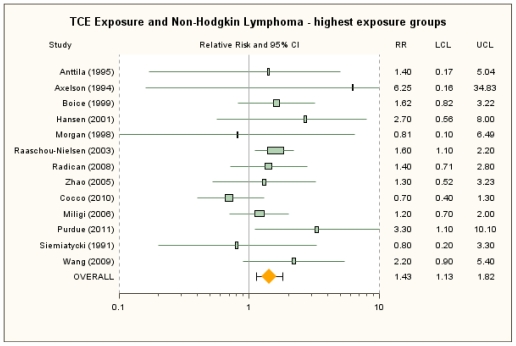
Forest plot of highest TCE exposure group and non-Hodgkin lymphoma from random-effects meta-analysis. Individual study results are plotted with 95% confidence intervals. Symbol sizes reflect relative weights of the 13 cohort and case-control studies.

The RRm estimate from the primary random-effects meta-analysis of the highest exposure groups from the 13 studies with results presented by exposure level was 1.43 (95% CI: 1.13, 1.82) (see Figure 6). No single study was overly influential; removal of individual studies resulted in RRm estimates that ranged from 1.38 (removing Purdue *et al*. [22]) to 1.57 (removing Cocco *et al*. [21]). Similarly, use of the nine alternative RR selections produced RRm estimates in a narrow range from 1.40 (95% CI: 1.09, 1.80) to 1.49 (95% CI: 1.14, 1.93) (see Supplemental Material). Low heterogeneity was observed across the 13 studies of NHL in the highest exposure groups (*I^2^* = 14%; *p* = 0.30), and low to low-to-moderate heterogeneity was apparent in each of the meta-analyses with alternative RR selections (*I^2^* = 9% to 33%; *p* = 0.12 to 0.37). Subgroup analyses examined the highest exposure groups in cohort and case-control studies, separately, to investigate the heterogeneity. In cohort studies, the RRm was 1.60 (95% CI: 1.24, 2.08) and, for case-control studies, 1.29 (95% CI: 0.76, 2.20). The subgroup RRm estimates were not statistically significantly different (*p* = 0.47, under the random-effects model).

## 4. Discussion

Individual studies in our analysis had low power to evaluate TCE exposure and cancer risk, but meta-analysis provided a tool to combine underpowered studies and to systematically assess the associations of TCE exposure and various cancers. In addition to the analyses of overall exposure, results were combined across the highest exposure groups, although individual studies used different exposure definitions, as an attempt to identify subjects with the greatest exposure potential and reduce potential exposure misclassification problems. We were unable to explore the shape of exposure-response relationships, as was recently done for benzene [61], given the few studies with quantitative TCE exposure data.

For kidney cancer, the elevated RRm estimates for overall TCE exposure and the highest exposure groups in the primary and alternative analyses provide robust support for a small, statistically significant increased risk, without evidence of heterogeneity or publication bias. The lack of observed heterogeneity provides evidence of consistency in kidney cancer risk estimates from independent epidemiologic studies of different industries with high potential for TCE exposure, regardless of study design. We did observe a slightly larger RRm estimate for case-control than for cohort studies; however, the difference across study designs was not statistically significant. Two case-control studies were carried out in geographic areas with a high frequency and a high degree of TCE exposure and were designed with *a priori* hypotheses to test for the effects of TCE exposure on renal cell cancer risk [49,50], and a third study was carried out in four central and eastern European countries with high renal cell cancer rates unexplained by established risk factors [24]. The higher exposures in these case-control studies compared to cohort studies may, in part, contribute to our finding of a larger RRm estimate for case-control studies.

We also found support for a small increased risk of NHL from TCE exposure, but less support than that for kidney cancer. Some potential publication bias in the TCE NHL data set was suggested by some of the tests used. In addition, low-to-moderate heterogeneity was observed for the NHL studies, although it was not statistically significant. Subgroup analysis of the cohort and case-control studies separately explained some of the differences in NHL risk across studies, although study design, itself, is unlikely to be an underlying cause of heterogeneity and is probably a surrogate for some other differences across these studies that may be associated with study design. Instead, temporal NHL classification changes and study differences in NHL classification, as well as differences in exposure definitions, levels, and prevalence, are possible alternative explanations. If TCE exposure increases the risk of NHL, the effects should be more apparent in the highest exposure groups and, indeed, our analyses did observe this finding. Our observations, furthermore, suggest an increased risk of liver cancer. The liver cancer results, however, are relatively underpowered with respect to numbers of studies and cases, and the study that provides the greatest weight used the weak exposure surrogate of duration of employment for categorizing exposure level.

Our analysis approach has several advantages to previous meta-analyses of TCE exposure and cancer. The selection criteria adopted for this meta-analysis were intended to identify informative studies for the evaluation of TCE exposure and cancer, *i.e.*, studies with reduced systematic errors. Neither Henschler *et al*. [62] nor Vamvakas *et al*. [63], two studies with incomplete cohort identification or potential selection bias of study controls, met our inclusion criteria. Their inclusion in another recent meta-analysis may have contributed to the observed heterogeneity in kidney cancer RR estimates in that analysis [16]. Despite these and other differences in study selection, such as the inclusion of studies lacking documented TCE exposure, that meta-analysis reported RRm estimates of 1.24 (95% CI: 1.06, 1.45) to 1.42 (95% CI: 1.13, 1.77) for overall exposure and kidney cancer [16], similar to our finding. Studies with low potential for TCE exposure also did not meet our selection criteria, as our analysis focused on studies in which TCE exposure potential was inferred to each subject by reference to industrial hygiene records, individual biomarkers, job-exposure matrices, or questionnaire responses, in order to reduce exposure misclassification bias, although this bias would not have been completely eliminated. Inclusion of studies of lower exposure potential in meta-analyses can have important implications for identifying a cancer hazard [64–66]. Additionally, we allowed use of a broader definition of NHL, more consistent with the updated WHO classification [67]. Use of this broader definition of NHL may have introduced some downward bias, since NHL subtypes unrelated to TCE may have been included. Last, our analysis updates other meta-analyses by the inclusion of recently published studies on NHL and kidney cancer, and we also included two studies of overlapping cohorts in alternate analyses. Two previous meta-analyses of TCE and NHL [18] or kidney cancer [16] chose to include Boice *et al*. [26] rather than Zhao *et al*. [25]. Despite the fact that Zhao *et al*. [25] did not report NHL results and we had to use results for a broader category of lymphohematopoietic cancers that included NHL, we preferred Zhao *et al*. [25] for our primary analysis because of their use of a semi-quantitative cumulative exposure assessment approach, use of an internal referent population, and much larger number of lymphohematopoietic cancer cases, which include NHLs, than Boice *et al*. [26] Our sensitivity analysis showed this study choice did not greatly affect the summary RRm for NHL and overall TCE exposure.

Interpretation of our findings on kidney cancer, liver cancer and NHL within a causal framework can be challenging in light of the modest RRm estimates and, for NHL, the low-to-moderate heterogeneity and potential publication bias. In general, the observed RRm estimates for overall TCE exposure suggest increased risks of 20%–30% for liver cancer, kidney cancer, and NHL. Increased risks suggested by the RRm estimates for the highest exposure groups were further elevated for kidney cancer (58%) and NHL (43%). Large RR estimates are considered strong evidence of causality; however, modest-sized risks may reflect a lower level of exposure or an agent of lower potency.

Consideration of potential confounding as an alternative explanation for our observations is important. Obesity, high body mass index (BMI), and smoking are known risk factors for kidney cancer [68]. Any confounding in cohort studies related to obesity is likely small given the generally healthy nature of an employed population. All kidney cancer case-control studies controlled for BMI, except Pesch *et al*. [52] and Moore *et al*. [24], and for smoking, except Moore *et al*. [24]. Moore *et al*. [24] reported that neither smoking nor BMI significantly changed the overall association with TCE exposure. Information on smoking for individual subjects is commonly lacking in cohort studies. Use of internal controls, which occurred in five of the cohort studies [25–27,41,43], generally minimizes effects of potential confounding due to smoking or socioeconomic status, since exposed and referent subjects are drawn from the same target population. Unlike for kidney cancer, a pattern of increased lung cancer risk was not apparent in the cohort studies; the RRm for lung cancer from the nine cohort studies in our meta-analysis was 0.96 (95% CI: 0.76, 1.21) for overall TCE exposure and 0.96 (95% CI: 0.72, 1.27) for the highest exposure groups. Although smoking was more prevalent in the Raaschou-Nielsen *et al.* [40] cohort than in the background population, if smoking fully explains the observed 40% excess lung cancer risk in this study, the expected contribution to renal cell carcinoma risk from smoking based on RRs for lung cancer and kidney cancer observed in five smoking cohorts [69] is estimated as 1%–6%, far smaller than the 20% and 40% excess in renal cell carcinoma risk observed in the cohort and subcohort, respectively. These observations suggest that confounding by smoking is not an issue for the kidney cancer results.

Job titles such as a degreaser often have potential for several exposures, including mineral oils, hydrazine, and other solvents, besides TCE [25,27,38,41]. Mineral oils such as cutting fluids, common to some job titles with potential TCE exposures, were included as covariates in the statistical analyses of Zhao *et al*. [25], who also examined hydrazine and several other co-exposures, and of Charbotel *et al*. [50,70] or were evaluated as a single exposure for cases and controls in some other studies [49,71]. Although Brüning *et al*. [49] reported an association with cutting oil exposure and kidney cancer, cutting oil exposure did not appear highly correlated with TCE exposure, as only 5 cases reported exposure to cutting oils compared to 25 cases reporting TCE exposure. Karami *et al*. [71], who examined mineral oil or cutting fluid exposure among cases and controls in Moore *et al*. [24], found no association for cutting oil mists or other mineral oil mists and kidney cancer. Cutting oils and mineral oils have not been associated with kidney cancer in other cohort or case-control studies [72,73]. Potential co-exposure to other solvents and other chemicals is unlikely to provide an alternative explanation for our robust findings, as the studies included in our analysis varied in the pattern, level, and specific types of co-exposures.

Risk factors for liver cancer include Hepatitis C viruses and heavy alcohol consumption in the United States and Northern Europe, where Hepatitis B prevalence is low [74]. In addition, nonalcoholic steatohepatitis, reflecting obesity and metabolic syndrome, was recently identified as contributing to liver cancer risk [68,75]. Heavy alcohol consumption is unlikely a confounder for liver cancer, as four of the nine independent cohort studies also reported on cirrhosis mortality, with no observed positive association with TCE exposure [26,27,41,43]. The generally healthy nature of an employed population reduces concern about confounding related to obesity and Hepatitis C.

Few risk factors have been identified for NHL, with the exception of viruses and suspected factors such as family history of NHL and lymphoproliferative diseases or immunosuppression [76]. Smoking is weakly associated with the follicular type of NHL [68,77]; however, any potential confounding by smoking in the case-control studies is reduced by the inclusion of several NHL types in our definition. Altered immunity may be a possible mode of action for TCE and NHL, as Lan *et al*. [78] recently reported decreased lymphocyte subsets among TCE-exposed workers.

In conclusion, our analysis updates the literature review since past meta-analyses on TCE exposure and NHL, liver cancer and kidney cancer, adopting criteria to identify studies that minimize biases associated with exposure misclassification and subject selection. The consistency of increased kidney cancer RR estimates across a large number of independent studies of different designs and populations from different countries and industries and the robust summary RRm estimates across various influence and sensitivity analyses provide strong support for a causal association. Although the RRm for kidney cancer was modest, neither chance nor confounding related to BMI, smoking, or exposure to cutting oils could provide alternative explanations for the observed increase in risk for this site. The support is strong but less robust for NHL, where issues of (non-statistically significant) study heterogeneity, potential publication bias, and weaker exposure-response results contribute greater uncertainty, and more limited for liver cancer, where only cohort studies with small numbers of cases are available. Although we did not examine exposure-response relationships using statistical models, biological gradients are supported for kidney cancer and NHL based on meta-analyses of only the highest exposure groups, which yielded higher summary RRm estimates than for overall TCE exposure. Other human, animal and pharmacokinetic data linking TCE and these cancers provide further support and biological plausibility to our findings [60].

## Supplementary Material



## Figures and Tables

**Table 1:** Key characteristics of epidemiologic cohort and case-control studies of TCE exposure included in the meta-analysis.

Reference	Study Design	Study population and size		Outcome and Sites Examined	Exposure assessment and exposure surrogate
Anttila *et al*. [38]	C	Finnish workers (n = 3,974) biologically monitored using U-TCA (n = 3,089), 1965–1982, FU 1965–1991 (M), 1967–1992 (I).	I, M	K, L, NHL	Subjects from several industries, primarily metal. Using the Ikeda *et al*. [54] relationship for TCE exposure to U-TCA, TCE exposures were roughly 4 ppm (median) and 6 ppm (mean). Overall TCE exposure, mean U-TCA, years since 1st U-TCA measurement.
Axelson *et al*. [37]	C	Swedish workers biologically monitored using U-TCA (n = 1,670), 1955–1975, FU 1958–1987 (I).	I	K, L, NHL	Roughly ¾ of cohort had U-TCA concentrations equivalent to <20 ppm TCE. Overall TCE exposure mean U-TCA, years since 1st biological monitoring measurement.
Boice *et al*. [41]	C	Aircraft-manufacturing workers with ≥1 year at Lockheed Martin (Burbank, CA) (n = 77,965; 2,267 with routine TCE; 3,176 with intermittent TCE), FU 1960–1996.	M	K, L, NHL	TCE subcohort. JEM for potential TCE exposure for (1) routine or (2) intermittent or routine basis without semi-quantitative intensity estimate. Overall TCE exposure, exposure duration.
Boice *et al*. [26]	C	Aerospace workers with ≥6 months employment at Rockwell/ Rocketdyne (Santa Susana Field Laboratory and nearby facilities) (n = 41,351; 1,111 with TCE exposure), FU 1948–1999. Overlaps cohort of Zhao *et al*. [25].	M	K, L, NHL	TCE subcohort. Potential TCE exposure assigned to test-stand workers whose tasks included the cleaning or flushing of rocket engines (engine flush) (*n* = 639 subjects) or for general utility cleaning (*n* = 472). JEM for TCE without semi-quantitative intensity estimates. Vapor degreasing with TCE before 1966 and PCE afterwards. Overall TCE exposure, exposure duration.
Brüning *et al*. [49]	CC	Histologically confirmed RCC (n = 134), from hospitals (Arnsberg, Germany), 1992−2000; hospital controls (n = 401).	I	K (RCC)	Self-reported exposure and JEM of Pannett *et al*. [55] to assign cumulative exposure to TCE and PCE. Cumulative exposure, exposure duration.
Charbotel *et al*. [50]	CC	RCC (n = 87), from urologists’ files and area teaching hospitals (Arve Valley region, France), 1993–2003; urologist or general practitioner patient controls (n = 316).	I	K (RCC)	Semi-quantitative cumulative TCE exposure and presence/absence of peak TCE exposure assigned to subjects using a JTEM designed using information obtained from questionnaires and routine atmospheric monitoring of workshops or biological monitoring (U-TCA) of workers carried out since the 1960s. Cumulative exposure (low, 62.4 ppm-year; medium, 253.2 ppm-year; high, 925.0 ppm-year), cumulative exposure + peaks.
Cocco *et al*. [21]	CC	Histologically confirmed NHL from 7 European countries (Czech Republic, Finland, France, Germany, Ireland, Italy, and Spain) (n = 2,348), 1998−2004; hospital (4 participating countries) or population controls (all others) (n = 2,462).	I	NHL	IH assessment of 43 agents, including TCE, by confidence, exposure intensity, and exposure frequency, in each participating center. Overall TCE exposure, cumulative TCE exposure for subjects assessed with high degree of confidence.
Dosemeci *et al*. [51]	CC	Histologically confirmed RCC (n = 438), 1988−1990, Minnesota Cancer Registry; controls identified using RDD or, if ≥65 years, from HCFA records (n = 687).	I	K (RCC)	Occupational history of TCE exposure using job title and JEM of Gomez *et al*. [56]. Overall TCE exposure.
Greenland *et al*. [42]	Nested CC	Cancer deaths among pensioned workers, GE transformer plant (Pittsfield, MA) (n = 12 kidney, 9 liver and biliary, 15 NHL), 1969–1984; controls were non-cancer deaths among pensioned workers (n = 1,202).	M	K, L, NHL	IH assessment from interviews and position descriptions. TCE (no/any exposure) assigned to individual subjects using JEM. Overall TCE exposure.
Hansen *et al*. [39]	C	Workers biologically monitored using U-TCA and air-TCE (n = 803), 1947–1989, FU 1968–1998.	I	K, L, NHL	U-TCA from 1947−1989; air TCE measurements from 1974. Overall, TCE exposure to cohort as extrapolated from air TCE and U-TCA measurements, using Ikeda *et al*. [54], was 4 ppm (median) and 12 ppm (mean). Overall TCE exposure, year 1st employed, employment duration, mean exposure, cumulative exposure.
Hardell *et al*. [46]	CC	Histologically confirmed cases of NHL in males from Swedish (Umea) hospital (n = 105), 1974−1978; population controls or, if case deceased, from causes-of-death registry (n = 335).	I	NHL	Self-reported overall TCE exposure.
Miligi *et al*. [20]	CC	NHL, including CLL, cases (n = 1,428) identified through surveys of hospital and pathology departments or specialized hematology centers in 8 areas in Italy, 1991−1993; population controls (n = 1,530).	I	NHL+ CLL	TCE exposure assigned using JEM and assessed using exposure probability, intensity and duration. TCE exposure intensity, exposure duration.
Moore *et al*. [24]	CC	Histologically confirmed RCC identified in hospitals in four European countries (Czech Republic, Poland, Romania, Russia) (n = 1,097), 1999−2003; hospital controls with diagnoses unrelated to smoking or genitourinary disorders (n = 1,476).	I	K (RCC)	Specialized job-specific questionnaire for specific jobs or industries of interest focused on solvent exposures, includingTCE, with exposure assignment by frequency, intensity and confidence of TCE exposure. Overall TCE exposure, duration (total hours, years), cumulative exposure (cases: 0, 0.83, 1.95, 7.25 ppm-years for 25th percentile, median, and 75th percentile) and average intensity (cases: 0, 0.08, 0.08, and 0.44 ppm for 25th percentile, median, and 75th percentile).
Morgan *et al*. [43]	C	Aerospace workers with >6 months during 1950–1985 at Hughes (Tucson, AZ) (n = 20,503; 4,733 with TCE exposure), FU 1950–1993.	M	K, L, NHL	TCE subcohort. TCE exposure intensity assigned using JEM. “High TCE exposure” job classification defined as >50 ppm. Overall TCE exposure, cumulative exposure, peak exposure.
Nordstrom *et al*. [47]	CC	Histologically confirmed cases of hairy-cell leukemia in males (n = 111), Swedish Cancer Registry, 1987−1992;^a^ population controls (n = 400).	I	NHL (HCL)	Self-reported overall TCE exposure.
Persson and Fredrikson [48]	CC	Histologically confirmed B-cell NHL from two hospitals in Sweden: Oreboro, 1964−1986, or Linkoping, 1975−1984 (n = 199); controls from previous studies, randomly selected from population registers (n = 479).	I	NHL	Self-reported overall TCE exposure.
Pesch *et al*. [52]	CC	Histologically confirmed RCC from German hospitals (5 regions) (n = 935), 1991−1995; controls randomly selected from residency registries (n = 4,298).	I	K (RCC)	TCE and other exposures assigned by questionnaire assessing occupational history using job title (JEM approach), job task (JTEM approach), or self-reported exposure. Cumulative exposure.
Purdue *et al*. [22]	CC	Histologically confirmed NHL identified from four SEER areas (Los Angeles County, Detroit metropolitan area, Seattle-Puget Sound and Iowa) (n = 1,321), 1998–2000; population controls from RDD, or Medicare file, if ≥65 years (n = 1,057).	I	NHL	Specialized job-specific modules asked for detailed information on individual jobs and focused on solvent exposures, including TCE; assessment by expert industrial hygienist blinded to case and control status by levels of probability, frequency, and intensity. Overall exposure, average weekly exposure, years exposed, average exposure intensity, and cumulative exposure. Cumulative exposure categories of 0, 1–46,800 ppm-hour, 46,801–112,320 ppm-hour, 112,321–234,000 ppmhour and >234,000 ppm-hour.
Radican *et al*. [27] (mortality follow-up of Blair *et al*. [44])	C	Civilian aircraft-maintenance workers with at least 1 year in 1952−1956 at Hill Air Force Base (UT) (n = 14,455; 7,204 ever exposed to TCE), FU 1952–2000 (M), 1973–1990 (I).	I, M	K, L, NHL	TCE assigned to individual subjects using JEM. TCE replaced in 1968 with 1,1,1-trichloroethane in bench-top degreasing activities and was discontinued in 1978 in vapor degreasing activities. Median TCE exposures were ~10 ppm for rag and bucket; 100−200 ppm for vapor degreasing. Overall TCE exposure, cumulative exposure, continuous or intermittent exposures, and peak exposure. Cumulative exposure categories of 0–5 unit-hour, 5–25 unit-hour, and >25 unit-hour.
Raaschou-Nielsen *et al*. [40]	C	Blue-collar workers employed >1,968 at 347 Danish TCE-using companies (n = 40,049; 14,360 with presumably higher-level exposure to TCE). FU to 1997.	I	K, L, NHL	Employers had documented TCE usage but no information on individual subjects, with job type and company size as variables identified as increasing the likelihood for TCE exposure. Subjects from iron and metal, electronics, painting, printing, chemical, and dry-cleaning industries. Median exposures to TCE were 40−60 ppm for the years before 1970, 10−20 ppm for 1970 to 1979, and approximately 4 ppm for 1980 to 1989. Overall TCE exposure, employment duration, year 1st employed, and company size.
Siemiatycki [53]	CC	Histologically confirmed NHL or kidney cancer in males, diagnosed in 16 large Montreal-area hospitals (215 = NHL, 177 K), 1979−1985; population-based controls identified from electoral lists and RDD (n = 533).	I	K, NHL	TCE and other exposure assigned on semi-quantitative scale from work histories by team of chemists and industrial hygienists. Overall TCE exposure, substantial exposure.
Wang *et al*. [23]	CC	Histologically confirmed NHL cases among females (n = 601), Connecticut Cancer Registry, 1996−2000; population-based female controls from RDD or, if ≥65 years of age, random selection from Medicare and Medicaid Service files (n = 717).	I	NHL	TCE intensity and probability of exposure assigned on semi-quantitative scale using JEM (Gomez *et al*. [56]). Overall TCE exposure, intensity, exposure probability.
Zhao *et al*. [25]	C	Aerospace workers with >2 years of employment at Rockwell/ Rocketdyne’s Santa Susana Field Laboratory, 1950–1993, FU 1950–2001 (M, n = 6,044), 1988–2000 (I, n = 5,049). Overlaps cohort of Boice *et al*.[26].	I, M	K, NHL+ other LHP cancers	TCE and other exposures assigned on semi-quantitative scale from work history using JEM. Cumulative TCE score.

a Upon a review of the case series, Nordstrom *et al*. [47] found one case was diagnosed in 1993.

C = cohort, CA = California, CC = case-control, CLL = chronic lymphocytic leukemia, FU = follow-up, HCFA = Health Care Financing Administration, HCL = hairy cell leukemia, I = incidence, IH = industrial hygiene, JEM = job-exposure matrix, JTEM = job-task-exposure matrix, K = kidney cancer, L = liver and biliary tract cancer, LHP = lymphohematopoietic, M = mortality, MA = Massachusettes, NHL = non-Hodgkin lymphoma, PCE = perchloroethylene, ppm = parts per million, RCC = renal cell carcinoma, RDD = random digit dialing, TCE = trichloroethylene, US = United States, U-TCA = urinary trichloroacetic acid, UT = Utah.

**Table 2:** RR estimates for kidney cancer associated with TCE exposure (overall and highest exposure group) from cohort and case-control studies.

	Overall Exposure		Highest TCE Exposure Group			
Study	RR (95% CI)	Alternate RR estimates	Exposure Category	RR (95% CI)	Alternate RR estimates	Comments
Cohort Studies						
Anttila *et al*. [38]	0.87 (0.32, 1.89)	None	100+ μmol/L U-TCA a	1.0 assumed b		ICD-7 180. SIR. Reported high exposure group results for some cancer sites but not kidney.
Axelson *et al*. [37]	1.16 (0.42, 2.52)	1.07 (0.39, 2.33) with estimated female contribution to SIR added^c^	≥2 year exposure and 100+ mg/L U-TCA	1.0 assumed b		ICD-7 180. SIR reported for males only, but there was a small female component to the cohort. Reported high exposure group results for some cancer sites but not kidney.
Boice *et al*. [41]	0.99 (0.4, 2.04)	None	≥5 years exposure	0.69 (0.22, 2.12)	None	ICD-9 189.0−189.2. Overall exposure SMR for potential routine exposure; results for any potential exposure not reported. Mortality RR for highest exposure group for potential routine or intermittent exposure, adjusted for date of birth, dates 1^st^ and last employed, race, and sex; referent group is workers not exposed to any solvent.
Greenland *et al*. [42]	0.99 (0.30, 3.32)	None	NA	b		ICD-8 codes not specified, presumably all of 189. Mortality OR from nested case-control study.
Hansen *et al*. [39]	1.1 (0.3, 2.8) c	None	≥1,080 months × mg/m^3^	1.0 assumed b		ICD-7 180. SIR. Reported high exposure group results for some cancer sites but not kidney.
Morgan *et al*. [43]	1.14 (0.51, 2.58) (Morgan *et al*. [57])	1.32 (0.57, 2.6) Published SMR	High cumulative exposure score	1.59 (0.68, 3.71)	1.89 (0.85, 4.23) for medium/high peak	ICD-7 180, ICD-8, -9 189.0−189.2. Overall mortality RR from Morgan *et al*. [57]. RRs adjusted for age and sex.
Raaschou-Nielsen *et al*. [40]	1.20 (0.94, 1.50)	1.20 (0.98, 1.46) for ICD-7 180; c 1.4 (1.0, 1.8) for subcohort with expected higher exposures	≥5 years in subcohort with expected higher exposure levels	1.7 (1.1, 2.4)	1.6 (1.1, 2.2) for ≥5 years in total cohort; c 1.4 (0.99, 1.9) ICD-7 180 ≥5 years in total cohort c	ICD-7 180.0 (RCC).
Radican *et al*. [27]	1.18 (0.47, 2.94)	None	>25 unit-years	1.11 (0.35, 3.49) d	Incidence RR: 0.9 (0.3, 3.2) (Blair *et al*. [44]) d	ICD-8, -9 189.0, ICD-10 C64. Mortality RR adjusted for age, sex and race, with workers with no chemical exposures as referent group.
Zhao *et al*. [25]	1.7 (0.38, 7.9)^e^	Incidence RR: 2.0 (0.47, 8.2); e Mortality RR no lag: 0.89 (0.22, 3.6); e Incidence RR no lag : 2.1 (0.56, 8.1);^e^ SMR: 2.22 (0.89, 4.57) (Boice *et al*. [26])	High exposure score	7.40 (0.47, 116)	Mortality RR: 1.82 (0.09, 38.6); Incidence RR no lag: 7.71 (0.65, 91.4); Mortality RR no lag: 0.96 (0.09, 9.91); Mortality RR: 2.12 (0.63, 7.11) for ≥5 years as test stand mechanic (Boice *et al*. [26]); 3.13 (0.74, 13.2) for ≥4 test-year engine flush (Boice *et al*. [26])	ICD-9 189. Mortality RR for males only for overall exposure with 20-year lag; adjusted for age, SES, time since first employment, exposure to other carcinogens. Overall mortality results reflect same number exposed cases (10 with no lag) as do incidence results. Overall RRs estimated by combining across exposure groups. Incidence RR for highest TCE exposure group reflects more exposed cases than does the mortality results and is used in primary analysis. Boice *et al.* [26] cohort, with seven exposed deaths, overlaps Zhao *et al.* [25] cohort.
Case-Control Studies: f						
Brüning *et al*. [58]	2.47 (1.36, 4.49)	1.80 (1.01, 3.20) for longest job held in industry with TCE exposure	≥20 years self-assessed exposure	2.69 (0.84, 8.66)	None	RCC. OR for self-assessed TCE exposure adjusted for age, sex, and smoking.
Charbotel *et al*. [50]	1.88 (0.89, 3.98)	1.64 (0.95, 2.84) for full study; 1.68 (0.97, 2.91) for full study with 10-year lag	High cumulative dose	3.34 (1.27, 8.74)	3.80 (1.27, 11.40) for high + peaks; Full study, high: 2.16 (1.02, 4.60) + peaks: 2.73 (1.06, 7.07); Full study with 10-year lag, high: 2.16 (1.01, 4.65) + peaks: 3.15 (1.19, 8.38); Full study, additional adjustment, high: 1.96 (0.71, 5.37) + peaks: 2.63 (0.79, 8.83)	RCC. ORs for subgroups with good confidence about exposure assessment. Matched on sex and age, and adjusted for smoking and BMI. Highest exposure group alternate estimates with additional adjustment were also adjusted for exposure to cutting fluids and other petroleum oils.
Dosemeci *et al*. [51]	1.30 (0.9, 1.9)	None	NA	b		RCC. OR adjusted for age, sex, smoking, hypertension and/or use of diuretics and/or anti-hypertension drugs, BMI.
Moore *et al*. [24]	2.05 (1.13, 3.73)	1.63 (1.04, 2.54) for all subjects	≥1.58 ppm × years	2.23 (1.07, 4.64)	2.02 (1.14, 3.59) for all subjects	RCC. Subgroup with high-confidence assessments. OR adjusted for age, sex, and center.
Pesch *et al*. [52]	1.24 (1.03, 1.49)	1.13 (0.98, 1.30) with German JEM	Substantial	1.4 (0.9, 2.1) d	1.2 (0.9, 1.7) for JEM d	RCC. JTEM approach. Crude ORs and CIs for overall TCE exposure calculated from data provided by Pesch [59], as described in methods section. ORs for highest exposure group adjusted for age, study center, and smoking.
Siemiatycki [53]	0.8 (0.3, 2.2)	None	Substantial	0.8 (0.2, 3.4)	None	“Kidney cancer.” SE and 95% CI calculated from reported 90% CI. OR for males only, adjusted for age, income, and cigarette smoking index.

a Mean personal trichloroacetic acid in urine. 1 μmol/L = 0.1634 mg/L.

b Anttila *et al*. [38], Axelson *et al*. [37] and Hansen *et al*. [39] report a RR estimate for highest TCE exposure groups and other cancers, but not kidney. A risk estimate of 1.0 is assigned for highest exposure in these studies to account for potential publication bias. For the SE (of the log RR) estimates for these null values, SE estimates from cancer types in the highest exposure group that were expected to have similar numbers of cases were generally used (See Appendix C of U.S. EPA [60] for further details). For Greenland *et al*. [42] and Dosemeci *et al*. [51], a risk of 1.0 is not assumed for highest exposure since only overall results are presented in those studies.

c Male and female results combined assuming Poisson distribution. Details of the approach used to estimate the female contribution for Axelson *et al*. [37] are presented in U.S. EPA [60].

d Male and female results combined using inverse-variance weighting, as in a fixed-effect meta-analysis.

e To derive an overall RR estimate, results were combined across exposure groups using inverse-variance weighting, under assumptions of group independence, although the exposure groups share a referent group and, hence, are not actually independent.

f The RR estimates are all ORs for incident cases.

BMI = body mass index, CI = confidence interval, cum = cumulative, ICD = International Classification of Diseases, JTEM = job-task-exposure matrix, NA = not available, OR = odds ratio, RCC = renal cell carcinoma, RR = relative risk, SE = standard error, SES = socioeconomic status, SIR = standardized incidence ratio, SMR = standardized mortality ratio, TCE = trichloroethylene, U-TCA = urinary trichloroacetic acid.

**Table 3:** Selected RR estimates for liver cancer associated with TCE exposure (overall and highest exposure group) from cohort and case-control studies.

	Overall Exposure		Highest TCE Exposure Group			
Study	RR (95% CI)	Alternate RR estimates	Exposure Category	RR (95% CI)	Alternate RR estimates	Comments
Cohort Studies						
Anttila *et al*. [38]	1.89 (0.86, 3.59) a	2.27 (0.74, 5.29) for 155.0 alone	100+ μmol/L U-TCA b	2.74 (0.33, 9.88)	None	ICD-7 155.0 + 155.1. SIR. ICD-7 155.0 for highest exposure group.
Axelson *et al*. [37]	1.41 (0.38, 3.60)	1.34 (0.36, 3.42) with estimated female contribution to SIR added c	100+ mg/L U-TCA	3.7 (0.09, 21) c	Exclude study	ICD-7 155. SIR reported for males only, but there was a small female component to the cohort. No cases were observed in highest exposure group (*i.e.*, >2 years and 100+ U-TCA), so combined with <2 years and 100+ subgroup and estimated female results.
Boice *et al*. [41]	0.81 (0.45, 1.33)	0.54 (0.15, 1.38) for potential routine exposure	≥5 year exposure	0.94 (0.36, 2.46)	None	ICD-9 155 + 156. Overall SMR for any potential exposure. Highest exposure mortality RR for any potential exposure, adjusted for date of birth, dates 1st and last employed, race, and sex; referent group is workers not exposed to any solvent.
Greenland *et al*. [42]	0.54 (0.11, 2.63)	None	NA	d		ICD-8 155 + 156. Mortality OR from nested case-control study.
Hansen *et al*. [39]	2.1 (0.7, 5.0) c	None	≥1,080 months × mg/m^3^	1.0 assumed d		ICD-7 155. SIR. Reported high exposure group results for some cancer sites but not liver.
Morgan *et al*. [43]	1.48 (0.56, 3.91)	0.98 (0.36, 2.13) Published SMR	High cumulative exposure score	1.19 (0.34, 4.16)	0.98 (0.29, 3.35) for medium/high	ICD-7 155, ICD-8, −9 155 + 156. Overall mortality RR as reported in Morgan *et al*. [57] RRs adjusted for age and sex.
Raaschou-Nielsen *et al*. [40]	1.35 (1.03, 1.77) a,c	1.28 (0.89, 1.80) for ICD-7 155.0 c	≥5 years	1.2 (0.7, 1.9) a,c	1.1 (0.5, 2.1) ICD-7 155.0 (liver only) c	ICD-7 155.0 + 155.1. SIR.
Radican *et al*. [27]	1.12 (0.57, 2.19)	1.25 (0.31, 4.97) for ICD-8, −9 155.0	>25 unit-year	1.49 (0.67, 3.34) e	None	ICD-8, −9 155 + 156, ICD-10 C22–C24. Mortality HR adjusted for age, sex and race, with workers with no chemical exposures as referent group.
Zhao *et al*. [25]/Boice *et al*. [26]	1.28 (0.35, 3.27)	1.0 assumed for Zhao *et al*. [25] d	High exposure score	1.0 assumed for Zhao *et al*. [25] d		ICD-9 155 + 156. Overall SMR for males from Boice *et al*. [26] used in lieu of Zhao *et al*. [25], who do not report liver cancer results. Highest exposure group RR for liver cancer not reported by Zhao *et al*. [25] or Boice *et al*. [26].

a Observed and expected numbers of cases combined assuming Poisson distribution for ICD codes identified in comments column.

b Mean personal trichloroacetic acid in urine. 1 μmol/L = 0.1634 mg/L.

c Male and female results combined assuming Poisson distribution. Details of the approach used to estimate the female contribution for Axelson *et al.* [37] are presented in U.S. EPA [60].

d Hansen *et al*. [39] and Zhao *et al*. [25] report a RR estimate for highest TCE exposure groups and other cancers, but not liver. A risk estimate of 1.0 is assigned for highest exposure in these studies, and as an alternate overall RR estimate for the Zhao *et al*. [25] study, which does not report any liver results, to account for potential publication bias. For the SE (of the log RR) estimates for these null values, SE estimates from cancer types that were expected to have similar numbers of cases were generally used (See Appendix C of U.S. EPA [60] for further details). For Greenland *et al*. [42], a risk of 1.0 is not assumed for highest exposure since only overall results are presented in that study.

e Male and female results combined using inverse-variance weighting, as in a fixed-effect meta-analysis.

HR = hazard ratio, ICD = International Classification of Diseases, NA = not available, OR = odds ratio, RR = relative risk, SIR = standardized incidence ratio, SMR = standardized mortality ratio, TCE = trichloroethylene, U-TCA = urinary trichloroacetic acid.

**Table 4:** Selected RR estimates for NHL associated with TCE exposure (overall and highest exposure group) from cohort and case-control studies.

	Overall Exposure		Highest TCE Exposure Group			
Study	RR (95% CI)	Alternate RR estimates	Exposure Category	RR (95% CI)	Alternate RR estimates	Comments
Cohort Studies						
Anttila *et al*. [38]	1.81 (0.78, 3.56)	None	100+ μmol/L U-TCA a	1.4 (0.17, 5.04)	None	ICD-7 200 + 202. SIR.
Axelson *et al*. [37]	1.52 (0.49, 3.53)	1.36 (0.44, 3.18) with estimated female contribution to SIR added b	≥2 year exposure and 100+ mg/L U-TCA	6.25 (0.16, 34.83)	5.62 (0.14, 31.3) with estimated female contribution added b	ICD-7 200 + 202. SIR reported for males only, but there was a small female component to the cohort.
Boice *et al*. [41]	1.19 (0.83, 1.65)	1.19 (0.65, 1.99) for potential routine exposure	≥5 years exposure	1.62 (0.82, 3.22)	None	ICD-9 200 + 202. Overall exposure SMR for any potential exposure. Mortality RR for highest exposure group for any potential exposure adjusted for date of birth, dates 1st and last employed, race, and sex; referent group is workers not exposed to any solvent.
Greenland *et al*. [42]	0.76 (0.24, 2.42)	None	NA			ICD-8 200–202. Mortality OR from nested case-control study. Overall exposure only.
Hansen *et al*. [39]	3.1 (1.3, 6.1) b	None	≥1,080 months × mg/m^3^	2.7 (0.56, 8.0) b	3.7 (1.0, 9.5) for >75 months exposure duration; b 2.9 (0.79, 7.5) for >19 mg/m^3^ mean exposure b	ICD-7 200 + 202. SIR for highest exposure group presented only for males; female results estimated and combined with male results.
Morgan *et al*. [43]	1.01 (0.46, 1.92)	1.36 (0.35, 5.21) RR for ICD 200	High cumulative exposure score	0.81 (0.1, 6.49)	1.31 (0.28, 6.08) for medium/high peak	ICD 200 + 202, ICD Revision 7, 8, or 9, depending on year of death. Overall SMR reported by Mandel *et al*. [18] Alternative overall mortality RR for ICD 200 as reported in Morgan *et al*. [57] and adjusted for age and sex. Mortality RR for highest exposure group is for ICD 200 only and adjusted for age and sex.
Raaschou-Nielsen *et al*. [40]	1.24 (1.01, 1.52)	1.5 (1.2, 2.0) for subcohort with expected higher exposures	≥5 years in subcohort with expected higher exposure levels	1.6 (1.1, 2.2)	1.45 (0.99, 2.05) for ≥5 years in full cohort b	ICD-7 200 + 202. SIR.
Radican *et al*. [27]	1.36 (0.77, 2.39)	None	>25 unit-years	1.41 (0.71, 2.81)^c^	0.97 (0.42, 2.2) for incidence (Blair *et al*. [44])^c^	ICD-8,-9 200 + 202; ICD-10 C82–C85. Mortality RR adjusted for age, sex and race, with workers with no chemical exposures as referent group.
Zhao *et al*. [25]	1.44 (0.90, 2.30) d	Incidence RR: 0.77 (0.42, 1.39); d SMR for ICD-9 200 + 202: 0.21 (0.01, 1.18) (Boice *et al*. [26])	High exposure score	1.30 (0.52, 3.23)	Incidence RR: 0.20 (0.03, 1.46)	Most lymphohematopoietic cancers, ICD-9 200–208, ICD-10, C81–C95, ICD-O 2, morphology code 9590–9716, 9723, 9800–9980. Mortality RRs used in primary analyses since reflect more exposed cases than do the incidence results. Males only; adjusted for age, SES, time since first employment.
Case-Control Studies:^e^						
Cocco *et al*. [21]	0.8 (0.5, 1.1)	None	High cumulative exposure	0.7 (0. 4, 1.3)	None	NHL. Grouping consistent with traditional definition provided by author. Incidence OR. High-confidence subgroup. Adjusted for age, sex, center, and education.
Hardell *et al*. [46]	7.2 (1.3, 42)	None	NA			NHL. Rappaport classification system. Incidence OR. Males only; controls matched for age, place of residence, vital status. Overall exposure only.
Miligi *et al*. [20]	0.93 (0.67, 1.29)	None	Medium/high exposure intensity	1.2 (0.7, 2.0)	1.0 (0.5, 2.6) for med/high intensity and >15 years	NHL + CLL. NCI Working Formulation. Adjusted OR for overall exposure not presented; overall crude incidence OR calculated as described in methods section. OR for highest exposure group adjusted for age, sex, education, and area.
Nordstrom *et al*. [47]	1.5 (0.7, 3.3)	None	NA			HCL. Incidence OR. Males only; controls matched for age and county; analysis controlled for age. Overall exposure only.
Perrson and Frederikson [48]	1.2 (0.5, 2.4)	None	NA			NHL. Classification system not specified. Incidence OR. Controls selected from same geographic areas; OR stratified on age and sex. Overall exposure only.
Purdue *et al*. [22]	1.4 (0.8, 2.4)	None	Cumulative exposure > 234,000 ppm-hours	3.3 (1.1, 10.1)	2.3 (1.0, 5.0) for highest exposure tertile (>112,320 ppm-hours)	ICD-O-3 codes 967–972. Incidence OR. Probable-exposure subgroup. Adjusted for age, sex, SEER center, race, and education.
Siemiatycki [53]	1.1 (0.5, 2.5)	None	Substantial	0.8 (0.2, 3.3)	None	ICD-9 200 + 202. Incidence OR. SE and 95% CI calculated from reported 90% CIs; males only; adjusted for age, income, and cigarette smoking index.
Wang *et al*. [23]	1.2 (0.9, 1.8)	None	Medium-high intensity	2.2 (0.9, 5.4)	None	ICD-O M-9590–9595, 9670–9688, 9690–9698, 9700–9723. Incidence OR. Females only; adjusted for age, family history of lymphohematopoietic cancers, alcohol consumption, and race.

a Mean personal trichloroacetic acid in urine. 1 μmol/L = 0.1634 mg/L.

b Male and female results combined assuming Poisson distribution. Details of the approach used to estimate the female contribution for Axelson *et al*. [37] are presented in U.S. EPA [60].

c Male and female results combined using inverse-variance weighting, as in a fixed-effect meta-analysis.

d To derive an overall RR estimate, results were combined across exposure groups using inverse-variance weighting, under assumptions of group independence, although the exposure groups share a referent group and, hence, are not actually independent.

e The RR estimates are all ORs for incident cases.

CI = confidence interval, CLL = chronic lymphocytic leukemia, HCL = hairy cell leukemia, ICD = International Classification of Diseases, NCI = National Cancer Institute, NHL = non-Hodgkin lymphoma, NA = not available, OR = odds ratio, RR = relative risk, SES = socioeconomic status, SIR = standardized incidence ratio, SMR = standardized mortality ratio, TCE = Trichloroethylene, U-TCA = urinary trichloroacetic acid.
