# Supplementary material for: Trichloroethylene and Cancer: Systematic and Quantitative Review of Epidemiologic Evidence for Identifying Hazards
Source: Int J Environ Res Public Health. 2011 Nov 9;8(11):4238–72. doi: 10.3390/ijerph8114238 (PMC3228569; doi:10.3390/ijerph8114238)
Supplement: Supplementary file 1 [file ijerph-08-04238-s001.doc]

*Supplementary Information*

**Trichloroethylene and Cancer: Systematic and Quantitative Review of Epidemiologic Evidence for Identifying Hazard**

**Cheryl Siegel Scott * and Jennifer Jinot**

National Center for Environmental Assessment, Office of Research and Development, United States Environmental Protection Agency, 1200 Pennsylvania Avenue, Washington, DC 20460, USA;
E-Mail: Jinot.Jennifer@epa.gov

* Author to whom correspondence should be addressed: E-Mail: Scott.Cheryl@epa.gov;

Tel: +1-703-347-8590; Fax: +1-703-347-8694.

Received: 22 September 2011; in revised form: 21 October 2011 / Accepted: 26 October 2011 / Published:

______________________________________________________________________________

**Supplemental Table 1.** Summary of rationales for study selection for meta-analysis.

| **Decision Outcome** | **Studies** | **Primary reason(s)** |
| --- | --- | --- |
| Studies Recommended for Meta-analysis: | | |
|  | Siemiatycki ; Axelson *et al*.; Hardell *et al*. ; Greenland *et al*. ; Anttila *et al*. ; Morgan *et al*. ; Nordstrom *et al*. ; Boice *et al*. ; Dosemeci *et al*. ; Persson and Fredriksson; Pesch *et al*. ; Hansen *et al*. ; Brüning *et al*. ; Raaschou-Nielsen *et al*. ;  Zhao *et al*. ; Miligi *et al*. ; Charbotel *et al*. ; Radican  *et al*. (Blair *et al*. incidence); Wang *et al*. ; Moore *et al*. ; Cocco *et al*. ; Purdue *et al*. | Analytical study designs of cohort or case-control approaches;  Evaluation of cancer incidence or cancer mortality;  Specifically identified TCE exposure potential to individual study subjects by reference to industrial hygiene records, individual biomarkers, job exposure matrices, water distribution models, or self-reports  (case-control studies);  Reported results for kidney cancer, liver cancer, or  non-Hodgkin lymphoma (NHL) with relative risk estimates and corresponding confidence intervals (or information to allow calculation). |

**Supplementary Table 1.** *Cont.*

| **Decision Outcome** | **Studies** | **Primary reason(s)** |
| --- | --- | --- |
| Studies Not Recommended for Meta-analysis: | | |
|  | ATSDR ; Clapp and Hoffman ; Cohn *et al*. | Weakness with respect to analytical study design (*i.e.*, geographic-based, ecological or proportional mortality ratio design). |
|  | Wilcosky *et al*. ; Isacson *et  al*. ; Shindell and Ulrich ; Garabrant *et al*. ; Shannon *et  al*. ; Blair *et al*. ; Costa *et al*. ; ADHS ; Mallin ; Aickin *et al*. ; Sinks *et al*. ; Vartiainen *et al*. ; Morgan and Cassady ; Lee *et al*. ;  Aickin ; Chang *et al*. ; Coyle *et al*. ; ATSDR ; Sung *et al*. | TCE exposure potential not assigned to individual subjects using job exposure matrix, individual biomarkers, water distribution models, or industrial hygiene data. |
|  | Lowengart *et al*. ; Fredriksson  *et al*. ; McKinney *et al*. ; Heineman *et al*. Siemiatycki *et  al*. ; Aronson *et al*. ; Fritchi and Siemiatycki ; Dumas *et  al*. ; Kernan *et al*. ;  Shu *et al*. ; Parent *et al*. ; Pesch *et al*. ; De Roos *et al*. ; Goldberg *et al*. ; Costas *et al*. ;  Krishnadasan *et al*. | Cancer incidence or mortality reported for cancers other than kidney, liver, or NHL. |
|  | Ritz | Subjects monitored for radiation exposure with likelihood for potential confounding;  Cancer mortality and TCE exposure not reported for kidney cancer and all hemato- and lymphopoietic cancer reported as broad category. |
|  | Henschler *et al*. | Incomplete identification of cohort and index kidney cancer cases included in case series |
|  | Vamvakas *et al*. | Control selection may not represent case series, with potential for selection bias. |

**Supplemental Table 2. Summary of some meta-analysis results for TCE (overall) and kidney cancer.**

| **Analysis** | **# of studies** | **Model** | **RRm** | **95% LCL** | **95% UCL** | **Heterogeneity** | **Comments** |
| --- | --- | --- | --- | --- | --- | --- | --- |
| All studies | 15 | Random | 1.27 | 1.13 | 1.43 | None obs  (fixed = random) | Statistical significance not dependent on single study. No apparent publication bias. |
|  |  | Fixed | 1.27 | 1.13 | 1.43 |  |  |
| Cohort | 9 | Random | 1.16 | 0.96 | 1.40 | None obs | Not significant difference between CC and cohort studies (*p* = 0.12). |
|  |  | Fixed | 1.16 | 0.96 | 1.40 |  | Not significant difference between CC and cohort studies (*p* = 0.19). |
| Case-control | 6 | Random | 1.48 | 1.15 | 1.91 | Not significant  (*p* = 0.14) |  |
|  |  | Fixed | 1.36 | 1.17 | 1.39 |  |  |
| Alternate RR selections ^a^ | 15 | Random | 1.27–1.28 | 1.13–1.14 | 1.42−1.43 | None obs | With 3 different alternates from  Zhao *et al*. . |
|  | 15 | Random | 1.29 | 1.15 | 1.45 | None obs | With Boice *et al*. rather than  Zhao *et al*. . |
|  | 15 | Random | 1.27 | 1.13 | 1.43 | None obs | With estimated female contribution to Axelson *et al*. . |
|  | 15 | Random | 1.28 | 1.14 | 1.43 | None obs | With Morgan *et al*. published SMR. |
|  | 15 | Random | 1.27 | 1.13 | 1.42 | None obs | With Raaschou-Nielsen *et al*. all kidney cancer. |
|  | 15 | Random | 1.32 | 1.17 | 1.49 | None obs | With Raaschou-Nielsen *et al*.  high-exposure subcohort. |

**Supplemental Table 2. *Cont.***

| **Analysis** | **# of studies** | **Model** | **RRm** | **95% LCL** | **95% UCL** | **Heterogeneity** | **Comments** |
| --- | --- | --- | --- | --- | --- | --- | --- |
|  | 15 | Random | 1.26 | 1.12 | 1.41 | None obs | With Brüning *et al*. [14] longest job held in industry with TCE. |
|  | 15 | Random | 1.28 | 1.14 | 1.43 | None obs | With Charbotel *et al*. [18] full study, with and without 10-year lag. |
|  | 15 | Random | 1.27 | 1.13 | 1.43 | None obs | With Moore *et al*. [22] full study. |
|  | 15 | Random | 1.21 | 1.09 | 1.34 | None obs | With Pesch *et al*. [12] JEM. |
| Highest exposure groups | 10 | Random | 1.64 | 1.31 | 2.04 | None obs |  |
|  | 13 | Random | 1.58 | 1.28 | 1.96 | None obs | Using RR = 1 for Anttila *et al*. [5], Axelson *et al*. [2], and Hansen *et al*. [13]. |
|  | 13 | Random | 1.47–1.60 | 1.20–1.29 | 1.79–1.98 | See Supplemental Table 3 | Using RR = 1 for Anttila *et al*. [5], Axelson *et al*. [2], and Hansen *et al*. [13] and various alternate RR selection results.a |

^a^ Changing the primary analysis by one alternate RR each time.

CC = case-control; JEM = job-exposure-matrix; LCL = lower confidence limit; obs = observable; RR = relative risk estimate; RRm = summary relative risk estimate; UCL = upper confidence limit.

**Supplemental Table 3. Summary of some meta-analysis results for TCE (highest exposure groups) and kidney cancer.**

| **Analysis** | **Model** | **RRm** | **95% LCL** | **95% UCL** | **Heterogeneity** | **Comments** |
| --- | --- | --- | --- | --- | --- | --- |
| Analysis based on reported results | Random | 1.64 | 1.31 | 2.04 | None obs  (fixed = random) |  |
| Primary analysis | Random | 1.58 | 1.28 | 1.96 | None obs | Includes assumed values for Anttila *et al*. , Axelson *et al*. , and Hansen *et al*. .  Statistical significance not dependent on single study. |
| Alternate RR selections ^a^ | Random | 1.57 | 1.27 | 1.95 | None obs | With Blair *et al*. incidence RR instead of Radican *et al*. mortality HR. |
|  | Random | 1.60 | 1.29 | 1.98 | None obs | With Morgan *et al*. peak metric. |
|  | Random | 1.47, 1.55 | 1.20, 1.25 | 1.80, 1.91 | None obs | With Raaschou-Nielsen *et al*. >5 years in total cohort for all kidney cancer and for RCC, respectively. |
|  | Random | 1.56−1.58 | 1.26−1.28 | 1.93−1.96 | None obs | With Zhao *et al*. incidence unlagged and mortality with and without lag. |
|  | Random | 1.58−1.59 | 1.28−1.29 | 1.95−1.96 | None obs | With Boice *et al*. alternate for  Zhao *et al*. . |
|  | Random | 1.59 | 1.29 | 1.95 | None obs | With Moore *et al*. full study. |
|  | Random | 1.54−1.58 | 1.24−1.27 | 1.90−1.95 | None obs | With Charbotel *et al*. high cumulative dose + peaks in subgroup; and high cumulative dose and high cumulative dose + peaks in full study with and without 10-year lag and with and without additional adjustment for exposure to cutting fluids and other petroleum oils. |
|  | Random | 1.47 | 1.20 | 1.79 | Not significant (*p* = 0.44) | With Pesch *et al*. JEM. |

^a^ Changing the primary analysis by one alternate RR each time.

HR = hazard ratio; JEM = job-exposure-matrix; LCL = lower confidence limit; obs = observable; RCC = renal cell carcinoma; RR = relative risk estimate; RRm = summary relative risk estimate; UCL = upper confidence limit.

**Supplemental Table 4.** Summary of some meta-analysis results for TCE and liver cancer.

| **Analysis** | **# of studies** | **Model** | **RRm** | **95% LCL** | **95% UCL** | **Heterogeneity** | **Comments** |
| --- | --- | --- | --- | --- | --- | --- | --- |
| All studies  (all cohort studies) | 9 | Random | 1.29 | 1.07 | 1.56 | None obs  (fixed = random) | Statistical significance not dependent on single study, except for  Raaschou-Nielsen *et al*. , without which *p* = 0.15. No apparent publication bias. |
|  |  | Fixed | 1.29 | 1.07 | 1.56 |  |  |
| All studies; liver cancer only, when available | 9 | Random | 1.25 | 0.99 | 1.57 | None obs | Used RR estimates for liver cancer alone for the 3 studies that presented these; remaining RR estimates are for liver and gall bladder/biliary passage cancers. |
| Alternate RR selections ^a^ | 9 | Random | 1.28 | 1.06 | 1.55 | None obs | With RR = 1 assumed for Zhao *et  al*. in lieu of Boice *et al*. . |
|  | 9 | Random | 1.34 | 1.09 | 1.63 | None obs | With Boice *et al*. potential routine exposure rather than any  potential exposure. |
|  | 9 | Random | 1.29 | 1.07 | 1.55 | None obs | With estimated female contribution to Axelson *et al*. . |
|  | 9 | Random | 1.26 | 1.05 | 1.52 | None obs | With Morgan *et al*. published SMR. |
| Highest exposure  groups | 6 | Random | 1.32 | 0.93 | 1.86 | None obs |  |
|  | 8 | Random | 1.28 | 0.93 | 1.77 | None obs | Primary analysis. Using RR = 1 for Hansen *et al*. and  Zhao *et al*. . |
|  | 7−8 | Random | 1.24−1.26 | 0.88−0.91 | 1.73−1.82 | None obs | Using alternate selections^a^ for Morgan *et al*. and  Raaschou-Nielsen *et al*. and excluding Axelson *et al*. . |

^a^ Changing the primary analysis by one alternate RR each time.

LCL = lower confidence limit; obs = observable; RR = relative risk estimate; RRm = summary relative risk estimate; SMR = standardized mortality ratio; UCL = upper confidence limit.

**Supplemental Table 5.** Summary of some meta-analysis results for TCE (overall) and NHL.

| **Analysis** | **# of studies** | **Model** | **RRm** | **95% LCL** | **95% UCL** | **Heterogeneity** | **Comments** |
| --- | --- | --- | --- | --- | --- | --- | --- |
| All studies | 17 | Random | 1.23 | 1.07 | 1.42 | Not significant  (*p* = 0.16)  *I^2^* = 26% | Statistical significance of RRm not dependent on individual studies. |
|  |  | Fixed | 1.21 | 1.08 | 1.35 |  |  |
| Cohort | 9 | Random | 1.33 | 1.13 | 1.58 | Not significant  (*p* = 0.34)  *I^2^* = 12% | Not significant difference between CC and cohort studies (*p* = 0.19). |
|  |  | Fixed | 1.31 | 1.14 | 1.51 |  | Not significant difference between CC and cohort studies (*p* = 0.08). |
| Case-control | 8 | Random | 1.11 | 0.89 | 1.38 | Not significant  (*p* = 0.22)  *I^2^* = 27% |  |
|  |  | Fixed | 1.07 | 0.90 | 1.28 |  |  |
| Alternate RR selections ^a^ | 17 | Random | 1.20 | 1.03 | 1.39 | Not significant  (*p* = 0.11)  *I^2^* = 31% | With estimated Zhao *et al*. overall RR for incidence rather than mortality. |
|  | 17 | Random | 1.22 | 1.03 | 1.43 | Not significant  (*p* = 0.09)  *I^2^* = 34% | With Boice *et al*. rather than Zhao *et al*. . |
|  | 17 | Random | 1.23 | 1.07 | 1.42 | Not significant  (*p* = 0.16)  *I^2^* = 25% | With estimated female contribution to Axelson *et al*. . |
|  | 17 | Random | 1.24 | 1.07 | 1.44 | Not significant  (*p* = 0.16)  *I^2^* = 26% | With Boice *et al*. potential routine exposure SMR. |

**Supplemental Table 5.** Summary of some meta-analysis results for TCE (overall) and NHL.

| **Analysis** | **# of studies** | **Model** | **RRm** | **95% LCL** | **95% UCL** | **Heterogeneity** | **Comments** |
| --- | --- | --- | --- | --- | --- | --- | --- |
|  | 17 | Random | 1.25 | 1.08 | 1.44 | Not significant  (p = 0.17)  I2 = 25% | With Morgan *et al*. [71] unpublished RR. |
|  | 17 | Random | 1.28 | 1.09 | 1.49 | Not significant  (p = 0.09)  I2 = 34% | With Raaschou-Nielsen *et al*. [15] subgroup expected to have higher exposures. |
| Alternate analysis; traditional definition of NHL only | 13 | Random | 1.27 | 1.05 | 1.55 | Not significant  (p = 0.054)  I2 = 42% | Omitting Miligi *et al*. [17],  Nordstrom *et al*. [7], Persson and Frederikson [11], and Greenland *et al*. [4], and including Boice *et al*. [8] instead of Zhao *et al*. [16]. |
| Highest exposure groups | 13 | Random | 1.43 | 1.13 | 1.82 | Not significant  (p = 0.30)  I2 = 14% | Statistical significance not dependent on single study. |
|  |  | Fixed | 1.43 | 1.16 | 1.75 |  |  |
|  |  | Random | 1.40−1.49 | 1.05−1.15 | 1.75−1.93 | See Supplemental Table 6. | Using alternative RR selection results ^a^ |

^a^ Changing the primary analysis by one alternate RR each time; more details on alternate RR estimates in text. CC = case-control; LCL = lower confidence limit; NHL = non-Hodgkin lymphoma; RR = relative risk estimate; RRm = summary relative risk estimate; SMR = standardized mortality ratio;
UCL = upper confidence limit.

**Supplemental Table 6. Summary of some meta-analysis results for TCE (highest exposure groups) and NHL.**

| **Analysis** | **# of studies** | **Model** | **Combined RR estimate** | **95% LCL** | **95% UCL** | **Heterogeneity** | **Comments** |
| --- | --- | --- | --- | --- | --- | --- | --- |
| All studies | 13 | Random | 1.43 | 1.13 | 1.82 | Not significant  (*p* = 0.30)  *I^2^* = 14% | Statistical significance not dependent on  single study. |
|  | 13 | Fixed | 1.43 | 1.16 | 1.75 |  |  |
| Cohort Studies | 8 | Random | 1.60 | 1.24 | 2.08 | None observable (random = fixed) | Not significant difference between CC and cohort studies (*p* = 0.47). |
|  | 8 | Fixed | 1.60 | 1.24 | 2.08 |  | Not significant difference between CC and cohort studies (*p* = 0.15). |
| Case-Control Studies | 5 | Random | 1.29 | 0.76 | 2.20 | NS (*p* = 0.08)  *I^2^* = 53% |  |
|  | 5 | Fixed | 1.18 | 0.84 | 1.64 |  |  |
| Alternate RR selections ^a^ | 13 | Random | 1.40 | 1.11 | 1.75 | NS (*p* = 0.33)  *I^2^* = 11% | With Raaschou-Nielsen *et al*. full cohort instead of subgroup expected to have  higher exposures. |
|  | 13 | Random | 1.40 | 1.09 | 1.80 | NS (*p* = 0.25)  *I^2^* = 19% | With Blair *et al*. incidence RR instead of Radican *et al*. mortality HR. |
|  | 13 | Random | 1.41 | 1.05 | 1.88 | NS (*p* = 0.12)  *I^2^* = 33% | With Zhao *et al*. incidence. |
|  | 13 | Random | 1.43 | 1.13 | 1.80 | NS (*p* = 0.32)  *I^2^* = 13% | With estimated female contribution for  Axelson *et al*. . |
|  | 13 | Random | 1.43 | 1.15 | 1.78 | NS (*p* = 0.37)  *I^2^* = 9% | With Purdue *et al*. highest cumulative exposure tertile. |
|  | 13 | Random | 1.44 | 1.12 | 1.85 | NS (*p* = 0.29)  *I^2^* = 16% | With Miligi *et al*. with >15 years. |

**Supplemental Table 6. Summary of some meta-analysis results for TCE (highest exposure groups) and NHL.**

| **Analysis** | **# of studies** | **Model** | **Combined RR estimate** | **95% LCL** | **95% UCL** | **Heterogeneity** | **Comments** |
| --- | --- | --- | --- | --- | --- | --- | --- |
|  | 13 | Random | 1.44 | 1.14 | 1.83 | NS (p = 0.32)  I2 = 13% | With Morgan *et al*. [6] peak. |
|  | 13 | Random | 1.45 | 1.14 | 1.86 | NS (p = 0.25)  I2 = 19% | With Hansen *et al*. [13] mean exposure. |
|  | 13 | Random | 1.49 | 1.14 | 1.93 | NS (p = 0.17)  I2 = 27% | With Hansen *et al*. [13] duration. |

^a^ Changing the primary analysis by one alternate RR estimate each time.

CC = case-control; HR = hazard ratio; LCL = lower confidence limit; NHL = non-Hodgkin lymphoma; NS = not statistically significant;
RR = relative risk estimate; RRm = summary relative risk estimate; UCL = upper confidence limit.

**Supplemental Figure 1.** Funnel plots of SE by log RR estimate for TCE and (**a**) kidney cancer, (**b**) liver cancer, and (**c**) NHL. Funnel plots were created using the software package Comprehensive Meta-Analysis, Version 2 (© 2006, Biostat, Inc.).

a.

**-2.0**

**-1.5**

**-1.0**

**-0.5**

**0.0**

**0.5**

**1.0**

**1.5**

**2.0**

**0.0**

**0.2**

**0.4**

**0.6**

**0.8**

**Standard Error**

**Log risk ratio**

**Funnel Plot of Standard Error by Log risk ratio**

b.

**Supplemental Figure 1.** *Cont.*

c.

**References**

1. Siemiatycki, J. *Risk Factors for Cancer in the Workplace*; CRC Press: Boca Raton, FL, USA, 1991.

2. Axelson, O.; Selden, A.; Andersson, K.; Hogstedt, C. Updated and expanded Swedish cohort study on trichloroethylene and cancer risk. *J. Occup. Med.* **1994**, *36*, 556-562.

3. Hardell, L.; Eriksson, M.; Degerman, A. Exposure to phenoxyacetic acids, chlorophenols, or organic solvents in relation to histopathology, stage, and anatomical localization of non-Hodgkin’s lymphoma. *Cancer Res.* **1994**, *54*, 2386-2389.

4. Greenland, S.; Salvan, A.; Wegman, D.H.; Hallock, M.F.; Smith, T.J. A case-control study of cancer mortality at a transformer-assembly facility. *Int. Arch. Occup. Environ. Health* **1994**, *66*, 49-54.

5. Anttila, A.; Pukkala, E.; Sallmen, M.; Hernberg, S.; Hemminki, K. Cancer incidence among Finnish workers exposed to halogenated hydrocarbons. *J. Occup. Environ. Med.* **1995**, *37*, 797-806.

6. Morgan, R.W.; Kelsh, M.A.; Zhao, K.; Heringer, S. Mortality of aerospace workers exposed to trichloroethylene. *Epidemiology* **1998**, *9*, 424-431.

7. Nordstrom, M.; Hardell, L.; Magnuson, A.; Hagberg, H.; Rask-Andersen, A. Occupational exposures, animal exposure and smoking as risk factors for hairy cell leukaemia evaluated in a
case-control study. *Br. J. Cancer* **1998**, *77*, 2048-2052.

8. Boice, J.D., Jr.; Marano, D.E.; Cohen, S.S.; Mumma, M.T.; Blot, W.J.; Brill, A.B.; Fryzek, J.P.; Henderson, B.E.; McLaughlin, J.K. Mortality among rocketdyne workers who tested rocket engines, 1948–1999. *J. Occup. Environ. Med.* **2006**, *48*, 1070-1092.

9. Boice, J.D., Jr.; Marano, D.E.; Fryzek, J.P.; Sadler, C.J.; McLaughlin, J.K. Mortality among aircraft manufacturing workers. *Occup. Environ. Med.* **1999**, *56*, 581-597.

10. Dosemeci, M.; Cocco, P.; Chow, W.H. Gender differences in risk of renal cell carcinoma and occupational exposures to chlorinated aliphatic hydrocarbons. *Am. J. Ind. Med.* **1999**, *36*, 54-59.

11. Persson, B.; Fredrikson, M. Some risk factors for non-Hodgkin’s lymphoma. *Int. J. Occup. Med. Environ. Health* **1999**, *12*, 135-142.

12. Pesch, B.; Haerting, J.; Ranft, U.; Klimpel, A.; Oelschlagel, B.; Schill, W. Occupational risk factors for renal cell carcinoma: Agent-specific results from a case-control study in Germany. MURC study group. Multicenter urothelial and renal cancer study. *Int. J. Epidemiol.* **2000**, *29*, 1014-1024.

13. Hansen, J.; Raaschou-Nielsen, O.; Christensen, J.M.; Johansen, I.; McLaughlin, J.K.; Lipworth, L.; Blot, W.J.; Olsen, J.H. Cancer incidence among Danish workers exposed to trichloroethylene. *J. Occup. Environ. Med.* **2001**, *43*, 133-139.

14. Bruning, T.; Pesch, B.; Wiesenhutter, B.; Rabstein, S.; Lammert, M.; Baumuller, A.; Bolt, H.M. Renal cell cancer risk and occupational exposure to trichloroethylene: Results of a consecutive
case-control study in Arnsberg, Germany. *Am. J. Ind. Med.* **2003**, *43*, 274-285.

15. Raaschou-Nielsen, O.; Hansen, J.; McLaughlin, J.K.; Kolstad, H.; Christensen, J.M.; Tarone, R.E.; Olsen, J.H. Cancer risk among workers at Danish companies using trichloroethylene: A cohort study. *Am. J. Epidemiol.* **2003**, *158*, 1182-1192.

16. Zhao, Y.; Krishnadasan, A.; Kennedy, N.; Morgenstern, H.; Ritz, B. Estimated effects of solvents and mineral oils on cancer incidence and mortality in a cohort of aerospace workers. *Am. J. Ind. Med.* **2005**, *48*, 249-258.

17. Miligi, L.; Costantini, A.S.; Benvenuti, A.; Kriebel, D.; Bolejack, V.; Tumino, R.; Ramazzotti, V.; Rodella, S.; Stagnaro, E.; Crosignani, P.; *et al.* Occupational exposure to solvents and the risk of lymphomas. *Epidemiology* **2006**, *17*, 552-561.

18. Charbotel, B.; Fevotte, J.; Hours, M.; Martin, J.L.; Bergeret, A. Case-control study on renal cell cancer and occupational exposure to trichloroethylene. Part II: Epidemiological aspects. *Ann. Occup. Hyg.* **2006**, *50*, 777-787.

19. Radican, L.; Blair, A.; Stewart, P.; Wartenberg, D. Mortality of aircraft maintenance workers exposed to trichloroethylene and other hydrocarbons and chemicals: Extended follow-up. *J. Occup. Environ. Med.* **2008**, *50*, 1306-1319.

20. Blair, A.; Hartge, P.; Stewart, P.A.; McAdams, M.; Lubin, J. Mortality and cancer incidence of aircraft maintenance workers exposed to trichloroethylene and other organic solvents and chemicals: Extended follow up. *Occup. Environ. Med.* **1998**, *55*, 161-171.

21. Wang, R.; Zhang, Y.; Lan, Q.; Holford, T.R.; Leaderer, B.; Zahm, S.H.; Boyle, P.; Dosemeci, M.; Rothman, N.; Zhu, Y.; *et al.* Occupational exposure to solvents and risk of non-Hodgkin lymphoma in Connecticut women. *Am. J. Epidemiol.* **2009**, *169*, 176-185.

22. Moore, L.E.; Boffetta, P.; Karami, S.; Brennan, P.; Stewart, P.S.; Hung, R.; Zaridze, D.;
Matveev, V.; Janout, V.; Kollarova, H.; *et al*. Occupational trichloroethylene exposure and renal carcinoma risk: Evidence of genetic susceptibility by reductive metabolism gene variants. *Cancer Res.* **2010**, *70*, 6527-6536.

23. Cocco, P.; t’Mannetje, A.; Fadda, D.; Melis, M.; Becker, N.; de Sanjose, S.; Foretova, L.;
Mareckova, J.; Staines, A.; Kleefeld, S.; *et al.* Occupational exposure to solvents and risk of lymphoma subtypes: Results from the Epilymph case-control study. *Occup. Environ. Med.* **2010**, *67*, 341-347.

24. Purdue, M.P.; Bakke, B.; Stewart, P.; De Roos, A.J.; Schenk, M.; Lynch, C.F.; Bernstein, L.; Morton, L.M.; Cerhan, J.R.; Severson, R.K.; *et al.* A case-control study of occupational exposure to trichloroethylene and non-Hodgkin lymphoma. *Environ. Health Perspect.* **2011**, *119*, 232-238.

25. Agency for Toxic Substances and Disease Registry (ATSDR). *Feasibility Investigation of Worker Exposure to Trichloroethylene at the View-Master Factory in Beaverton, Oregon*; ATSDR: Atlanta, GA, USA, 2004.

26. Clapp, R.W.; Hoffman, K. Cancer mortality in IBM Endicott plant workers, 1969–2001: An update on a NY production plant. *Environ. Health* **2008**, *7*, 13:1-13:4.

27. Cohn, P.; Klotz, J.; Bove, F.; Berkowitz, M.; Fagliano, J. Drinking water contamination and the incidence of leukemia and non-Hodgkin’s lymphoma. *Environ. Health Perspect.* **1994**, *102*, 556-561.

28. Wilcosky, T.C.; Checkoway, H.; Marshall, E.G.; Tyroler, H.A. Cancer mortality and solvent exposures in the rubber industry. *Am. Ind. Hyg. Assoc. J.* **1984**, *45*, 809-811.

29. Isacson, P.; Bean, J.A.; Splinter, R.; Olson, D.B.; Kohler, J. Drinking water and cancer incidence in Iowa. III. Association of cancer with indices of contamination. *Am. J. Epidemiol.* **1985**, *121*,
856-869.

30. Shindell, S.; Ulrich, S. A cohort study of employees of a manufacturing plant using trichlorethylene. *J. Occup. Med.* **1985**, *27*, 577-579.

31. Garabrant, D.H.; Held, J.; Langholz, B.; Bernstein, L. Mortality of aircraft manufacturing workers in southern California. *Am. J. Ind. Med.* **1988**, *13*, 683-694.

32. Shannon, H.S.; Haines, T.; Bernholz, C.; Julian, J.A.; Verma, D.K.; Jamieson, E.; Walsh, C. Cancer morbidity in lamp manufacturing workers. *Am. J. Ind. Med.* **1988**, *14*, 281-290.

33. Blair, A.; Haas, T.; Prosser, R.; Morrissette, M.; Blackman, K.; Grauman, D.; van, D.P.; Moran, F. Mortality among United States Coast Guard marine inspectors. *Arch. Environ. Health* **1989**, *44*,
150-156.

34. Costa, G.; Merletti, F.; Segnan, N. A mortality cohort study in a north Italian aircraft factory. *Br. J. Ind. Med.* **1989**, *46*, 738-743.

35. Arizona Department of Health Services (ADHS). *Update of the Incidence of Childhood Cancers and Testicular Cancer in Southwest Tuscon: 1987–1991*; ADHS: Phoenix, AZ, USA. 1995.

36. Arizona Department of Health Services (ADHS). *Incidence Study of Childhood Cancer in Maricopa County: 1965–1986*; ADHS: Phoenix, AZ, USA. 1990.

37. Mallin, K. Investigation of a bladder cancer cluster in northwestern Illinois. *Am. J. Epidemiol.* **1990**, *132*, S96-S106.

38. Aickin, M.; Chapin, C.A.; Flood, T.J.; Englender, S.J.; Caldwell, G.G. Assessment of the spatial occurrence of childhood leukaemia mortality using standardized rate ratios with a simple linear poisson model. *Int. J. Epidemiol.* **1992**, *21*, 649-655.

39. Sinks, T.; Lushniak, B.; Haussler, B.J.; Sniezek, J.; Deng, J.F.; Roper, P.; Dill, P.; Coates, R. Renal cell cancer among paperboard printing workers. *Epidemiology* **1992**, *3*, 483-489.

40. Vartiainen, T.; Pukkala, E.; Rienoja, T.; Strandman, T.; Kaksonen, K. Population exposure to trichloroethene and tetrachloroethene and cancer risk—Two cases of drinking-water pollution. *Chemosphere* **1993**, *27*, 1171-1181.

41. Morgan, J.W.; Cassady, R.E. Community cancer assessment in response to long-time exposure to perchlorate and trichloroethylene in drinking water. *J. Occup. Environ. Med.* **2002**, *44*, 616-621.

42. Lee, L.J.; Chung, C.W.; Ma, Y.C.; Wang, G.S.; Chen, P.C.; Hwang, Y.H.; Wang, J.D. Increased mortality odds ratio of male liver cancer in a community contaminated by chlorinated hydrocarbons in groundwater. *Occup. Environ. Med.* **2003**, *60*, 364-369.

43. Aickin, M. Bayes without priors. *J. Clin. Epidemiol.* **2004**, *57*, 4-13.

44. Chang, Y.M.; Tai, C.F.; Yang, S.C.; Chen, C.J.; Shih, T.S.; Lin, R.S.; Liou, S.H. A cohort mortality study of workers exposed to chlorinated organic solvents in Taiwan. *Ann. Epidemiol.* **2003**, *13*,
652-660.

45. Chang, Y.M.; Tai, C.F.; Yang, S.C.; Lin, R.S.; Sung, F.C.; Shih, T.S.; Liou, S.H. Cancer incidence among workers potentially exposed to chlorinated solvents in an electronics factory. *J. Occup. Health* **2005**, *47*, 171-180.

46. Coyle, Y.M.; Hynan, L.S.; Euhus, D.M.; Minhajuddin, A.T. An ecological study of the association of environmental chemicals on breast cancer incidence in Texas. *Breast Cancer Res. Treat.* **2005**, *92*, 107-114.

47. Agency for Toxic Substances and Disease Registry (ATSDR). *Health Consultation, Endicott Area Investigation, Cancer and Birth Outcome Analysis, Endicott area, Town of Union, Broome County, New York*; ATSDR: Atlanta, GA, USA. 2006.

48. Agency for Toxic Substances and Disease Registry (ATSDR). *Health Consultation, Health Statistics Review Follow-Up, Cancer and Birth Outcome Analysis, Endicott Area Investigation, Endicott Area, Town of Union, Broome County, New York*; ATSDR: Atlanta, GA, USA. 2008.

49. Sung, T.I.; Chen, P.C.; Jyuhn-Hsiarn Lee, L.; Lin, Y.P.; Hsieh, G.Y.; Wang, J.D. Increased standardized incidence ratio of breast cancer in female electronics workers. *BMC Public Health* **2007**, *7*, 102:1-102:9.

50. Sung, T.I.; Wang, J.D.; Chen, P.C. Increased risk of cancer in the offspring of female electronics workers. *Reprod. Toxicol.* **2008**, *25*, 115-119.

51. Lowengart, R.A.; Peters, J.M.; Cicioni, C.; Buckley, J.; Bernstein, L.; Preston-Martin, S.;
Rappaport, E. Childhood leukemia and parents’ occupational and home exposures. *J. Natl. Cancer Inst.* **1987**, *79*, 39-46.

52. Fredriksson, M.; Bengtsson, N.O.; Hardell, L.; Axelson, O. Colon cancer, physical activity, and occupational exposures. A case-control study. *Cancer* **1989**, *63*, 1838-1842.

53. McKinney, P.A.; Alexander, F.E.; Cartwright, R.A.; Parker, L. Parental occupations of children with leukaemia in West Cumbria, North Humberside, and Gateshead. *BMJ* **1991**, *302*, 681-687.

54. Heineman, E.F.; Cocco, P.; Gomez, M.R.; Dosemeci, M.; Stewart, P.A.; Hayes, R.B.; Zahm, S.H.; Thomas, T.L.; Blair, A. Occupational exposure to chlorinated aliphatic hydrocarbons and risk of astrocytic brain cancer. *Am. J. Ind. Med.* **1994**, *26*, 155-169.

55. Siemiatycki, J.; Dewar, R.; Nadon, L.; Gerin, M. Occupational risk factors for bladder cancer: Results from a case-control study in Montreal, Quebec, Canada. *Am. J. Epidemiol.* **1994**, *140*,
1061-1080.

56. Aronson, K.J.; Siemiatycki, J.; Dewar, R.; Gerin, M. Occupational risk factors for prostate cancer: Results from a case-control study in Montreal, Quebec, Canada. *Am. J. Epidemiol.* **1996**, *143*,
363-373.

57. Fritschi, L.; Siemiatycki, J. Melanoma and occupation: Results of a case-control study. *Occup. Environ. Med.* **1996**, *53*, 168-173.

58. Dumas, S.; Parent, M.E.; Siemiatycki, J.; Brisson, J. Rectal cancer and occupational risk factors: A hypothesis-generating, exposure-based case-control study. *Int. J. Cancer* **2000**, *87*, 874-879.

59. Kernan, G.J.; Ji, B.T.; Dosemeci, M.; Silverman, D.T.; Balbus, J.; Zahm, S.H. Occupational risk factors for pancreatic cancer: A case-control study based on death certificates from 24 U.S. states. *Am. J. Ind. Med.* **1999**, *36*, 260-270.

60. Shu, X.O.; Perentesis, J.P.; Wen, W.; Buckley, J.D.; Boyle, E.; Ross, J.A.; Robison, L.L. Parental exposure to medications and hydrocarbons and ras mutations in children with acute lymphoblastic leukemia: A report from the Children’s Oncology Group. *Cancer Epidemiol. Biomarkers Prev.* **2004**, *13*, 1230-1235.

61. Shu, X.O.; Potter, J.D.; Linet, M.S.; Severson, R.K.; Han, D.; Kersey, J.H.; Neglia, J.P.; Trigg, M.E.; Robison, L.L. Diagnostic X-rays and ultrasound exposure and risk of childhood acute lymphoblastic leukemia by immunophenotype. *Cancer Epidemiol. Biomarkers Prev.* **2002**, *11*, 177-185.

62. Parent, M.E.; Siemiatycki, J.; Fritschi, L. Workplace exposures and oesophageal cancer. *Occup. Environ. Med.* **2000**, *57*, 325-334.

63. Pesch, B.; Haerting, J.; Ranft, U.; Klimpel, A.; Oelschlagel, B.; Schill, W. Occupational risk factors for urothelial carcinoma: Agent-specific results from a case-control study in Germany. MURC study group. Multicenter urothelial and renal cancer. *Int. J. Epidemiol.* **2000**, *29*, 238-247.

64. De Roos, A.J.; Teschke, K.; Savitz, D.A.; Poole, C.; Grufferman, S.; Pollock, B.H.; Olshan, A.F. Parental occupational exposures to electromagnetic fields and radiation and the incidence of neuroblastoma in offspring. *Epidemiology* **2001**, *12*, 508-517.

65. Goldberg, M.S.; Parent, M.E.; Siemiatycki, J.; Desy, M.; Nadon, L.; Richardson, L.; Lakhani, R.; Latreille, B.; Valois, M.F. A case-control study of the relationship between the risk of colon cancer in men and exposures to occupational agents. *Am. J. Ind. Med.* **2001**, *39*, 531-546.

66. Costas, K.; Knorr, R.S.; Condon, S.K. A case-control study of childhood leukemia in Woburn, Massachusetts: The relationship between leukemia incidence and exposure to public drinking water. *Sci. Total Environ.* **2002**, *300*, 23-35.

67. Krishnadasan, A.; Kennedy, N.; Zhao, Y.; Morgenstern, H.; Ritz, B. Nested case-control study of occupational chemical exposures and prostate cancer in aerospace and radiation workers. *Am. J. Ind. Med.* **2007**, *50*, 383-390.

68. Ritz, B. Cancer mortality among workers exposed to chemicals during uranium processing. *J. Occup. Environ. Med.* **1999**, *41*, 556-566.

69. Henschler, D.; Vamvakas, S.; Lammert, M.; Dekant, W.; Kraus, B.; Thomas, B.; Ulm, K. Increased incidence of renal cell tumors in a cohort of cardboard workers exposed to trichloroethene. *Arch. Toxicol.* **1995**, *69*, 291-299.

70. Vamvakas, S.; Bruning, T.; Thomasson, B.; Lammert, M.; Baumuller, A.; Bolt, H.M.; Dekant, W.; Birner, G.; Henschler, D.; Ulm, K. Renal cell cancer correlated with occupational exposure to trichloroethene. *J. Cancer Res. Clin. Oncol.* **1998**, *124*, 374-382.

71. Morgan, R.W.; Kelsh, M.A.; Zhao, K.; Heringer, S. *Final Report “Cohort Mortality Study of Aerospace Workers with Exposure to Trichloroethylene.”*, *Letter from Paul A. Cammer, Ph.D., Trichloroethylene Issues Group, to Cheryl Siegel Scott, US EPA*, ID 645806; US EPA, Health and Environmental Reserach Online (HERO): Durham, NC, USA, 1997. Available online: http://hero.epa.gov/index.cfm?heroid=645806&action=search.do&submit=Search&portal=true&sort=year&all=&exact=&any=&author=&journal=&singleyear=&startyear=&endyear= (accessed on
30 August 2011).

© 2011 by the authors; licensee MDPI, Basel, Switzerland. This article is an open access article distributed under the terms and conditions of the Creative Commons Attribution license (http://creativecommons.org/licenses/by/3.0/).
